# Insertion attack identification in discrete event systems using petri nets with an observer

**DOI:** 10.1371/journal.pone.0314104

**Published:** 2024-12-09

**Authors:** Adeeb A. Ahmed, Yufeng Chen, Ahmed M. El-Sherbeeny

**Affiliations:** 1 Control Science and Engineering Department, School of Electro-Mechanical Engineering, Xidian University, Xi’an, China; 2 Industrial Engineering Department, College of Engineering, King Saud University, Riyadh, Saudi Arabia; Najran University College of Computer Science and Information Systems, SAUDI ARABIA

## Abstract

This study addresses the problem of attack identification in discrete event systems modeled with Petri nets, focusing specifically on sensor attacks that mislead observers to making incorrect decisions. Insertion attacks are one of the sensor attacks that are considered in this work. First, we formulate a novel observation structure to systematically model insertion attacks within the Petri net framework. Second, by generating an extended reachability graph that incorporates the observation structure, we can find a special class of markings whose components can have negative markings. Third, an observation place is computed by formulating an integer linear programming problem, enabling precise detection of attack occurrences. The occurrence of an attack can be identified by the number of tokens in the designed observation place. Finally, examples are provided to verify the proposed approach. Comparative analysis with existing techniques demonstrates that the reported approach offers enhanced detection accuracy and robustness, making it a significant advancement in the field of secure discrete event systems.

## 1 Introduction

Discrete event systems (DESs) evolve based on the occurrence of distinct discrete events. Their primary control objective is to adhere to desired specifications, such as deadlock prevention or liveness enforcement. As a technical abstraction, DESs are prevalent in cyber-physical systems(CPSs), especially in modern computer-integrated infrastructures. A discrete-state space with event-driven state transitions [1–4] distinctly marks them. The main mathematical tools for DES modeling are finite state automata(FSA) and Petri nets (PNs), and each can be used for different DES tasks [5, 6]. While PNs present a more compact system model, finite automata tend to enumerate system states explicitly.

**Fig 1:**
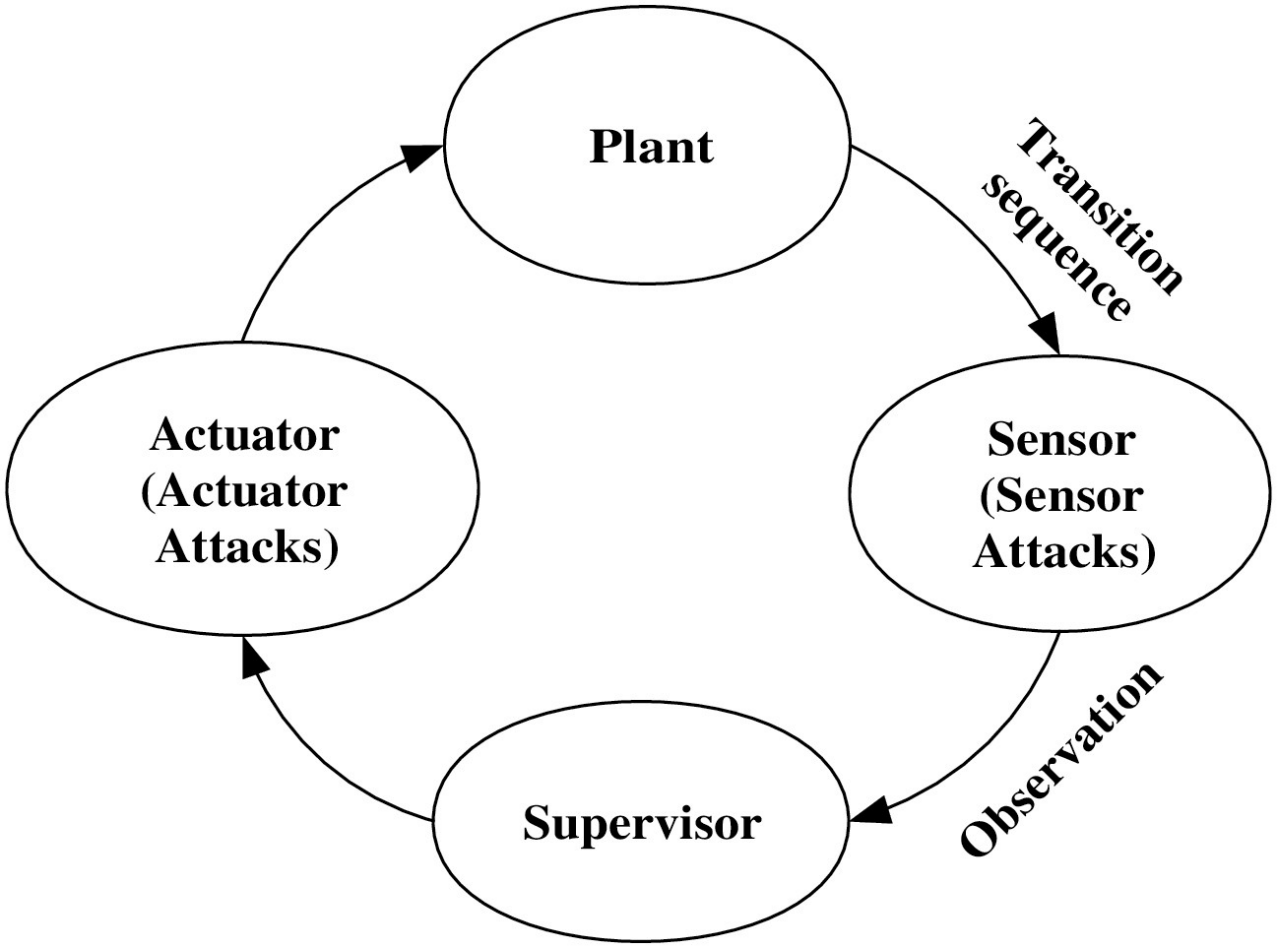
Closed-loop system under attacks.

The supervisory control theory offers a novel approach to DES control and has gained extensive applications [7, 8]. It aims to design controllers (or supervisors) to ensure that a DES satisfies predefined control specifications. The main problem in making these kinds of supervisors is the existence of events that cannot be controlled or seen, which is caused by the fact that DESs are inherently partially controllable and observable systems. It also influences related tasks, including fault diagnosis, diagnosability analysis, opacity verification, and detectability. Much research has been done on supervisory control [9], which needs a supervisor can change a system’s evolution by selectively disabling events based on real-time observations. Practically, this involves sensors monitoring events and actuators implementing supervisor directives. Fig 1 presents a closed-loop control system, delineating the interactions between the ‘Plant,’ ‘Sensor,’ ‘Supervisor,’ and ‘Actuator’. Notably, the system’s ‘Sensor’ and ‘Actuator’ components are vulnerable to attacks, raising security and control accuracy concerns.

As communication networks become increasingly integrated with closed-loop control systems, their susceptibility to network attacks intensifies. It increases the possibility of malicious entities manipulating sensor readings and control commands. Given the pivotal role of computer and network communication in the public security and education sectors, cyber-attack repercussions extend beyond economic damages. Thus, reevaluating supervisory control for DESs in light of potential attacks has become paramount [10, 11]. Numerous studies have focused on exploring supervisory control within DESs, particularly examining their vulnerability to attacks in scenarios characterized by nondeterministic observations [12, 13]. Adaptive supervisory control policies for automated manufacturing systems (AMS) with unreliable resources, ensuring deadlock-free operation and maintaining system performance during resource failures [14, 15]. Considering the challenges presented by partial observations, accurate state estimation in DESs becomes a crucial task, attracting significant interest among researchers specializing in PNs [16–20] and automata [21–24]. A substantial body of work addresses supervisory control in DESs subject to partial state estimations [25, 26].

Within the R-W framework, supervisory control amidst attacks has been presented [27], a concept inspired by prior research on linear time-invariant systems [28–30]. The authors of [27] discuss the challenge of an attacker corrupting the symbols perceived by a supervisor and consider the resilience of supervisors against malicious attacks. They emphasized the importance of controllability and a newly defined concept of observability for events within the original system. Expanding on the traditional observability introduced in [31, 32], they introduce a revised concept that considers potential interference from attackers. Also, the idea of stealthy attacks in a DES has been put forward, which cannot be fully seen. An attack structure is represented as a deterministic finite automaton. [33]. This structure ensures that an attacker can mislead a controller about the system’s state. Discerning if an external attack has targeted a system is a significant theoretical and practical issue. A detailed study [34] looks into how to find holes in DESs where an attacker could change the commands for controlling actuators and certain sensor readings. This work categorized attacks into four primary types and assessed supervisory control against these attacks, given a predefined control specification. Meanwhile, the work in [35] centered on discerning a control specification resilient against actuator channel attacks.

The challenges surrounding attack modeling and detection within DESs have garnered substantial attention in recent research. In [36], Fritz and Zhang propose an insightful method that explicitly addresses situations where all sensor and actuator channels are under deception attacks. These attacks, characterized by their elusive nature, cloak their system impact, making detection complex. An innovative measurement inconsistent DES framework is introduced in [37]. The research in [38] focuses on networked DESs that have control command delays. The work in [39] enhances the detection of multiplicative false data injection (FDI) attacks in process control systems through the use of randomized control mode switching. This method minimizes false alarms and prevents detection-evasive attacks, as demonstrated through simulations. The work in [40] investigates the nuances of multiple sensor attacks, which manipulate system observations using static or dynamic attack dictionaries. The research posits that a diagnostic structure is essential to solving the attack detection dilemma. A polynomial algorithm in [41] checks for sneaky sensor attackers by looking for possible control goal violations.

The study in [42] uses nondeterministic finite state transducers (NFST) to defend against many types of attacks. Building on the NFST model, this study addresses the supervisory control challenge in the presence of sensor and actuator attacks. Another important result [43] suggests a way to make a supreme, controllable, and strong supervisor that could protect against certain types of bounded sensor attacks. The novel bipartite transition structure introduced in [44] epitomizes the tug-of-war between the supervisor and attacker. This research thoroughly explores the synthesis of robust supervisors capable of resisting arbitrary sensor attacks without imposing any normal conditions. Furthermore, the PNs framework was used to see how resistant a supervisor is when attacked by sensors and actuators. We do this by checking how well each state fits the rules given by the reachability graph (RG) [45]. The study in [46] introduces data inhibitor arcs in PNs for optimal supervisory control. These arcs disable transitions based on token counts. The proposed method prevents illegal states and reduces supervisor complexity, achieving optimal control with minimal control places. The work in [47] proposes methods to estimate and prevent actuator enablement attacks within DESs under supervisory control, introducing the concepts of strong and weak AE-estimability and designing prevention modules to protect these systems without altering their original behaviors. In [48] the authors introduces an iterative method to convert nonlinear constraints into linear constraints for PNs. It separates admissible markings into subsets using linear constraints and designs a PN supervisor to enforce these constraints, ensuring system compliance and reducing complexity. The authors in [49] improves the efficiency of solving an integer linear programming problem (ILPP) for PN supervisors by reducing constraints and modifying supervisor structures, maintaining the same feasible solutions as existing methods.

According to a different study [50], CPSs need to protect against network attacks, especially sensor-reading modification attacks (SM-attacks). This study uses a bounded PN model and liveness-enforcing controls to devise a method that combats SM-attacks, ensuring consistent and accurate sensor readings within CPSs. In [51], supervisors work to prevent unsafe states despite limited visibility. Attackers can alter sensor data or enable blocked events to disrupt the system. An attack structure automaton aids attackers in choosing actions to jeopardize safety. The research in [52] focuses on identifying attacks in DESs and differentiating between sensor and actuator attacks. The study provides an algorithm to outline the system’s behavior during an attack and concludes with a complexity analysis.

In this manuscript, we present a comprehensive methodology for identifying sensor attacks in DESs modeled by PNs. This approach focuses on the main type of sensor attack, namely insertion attacks. To effectively model these attacks, we introduce a novel observation structure within the PN framework. This structure facilitates the generation of an extended reachability graph (ERG), enabling the identification of specific markings indicative of potential attacks. Central to the approach is the computation of an observation place through solving an ILPP. The detection of attacks is achieved by monitoring the token count within this observation place. The subsequent points outline the key contributions of this research:

1. We develop a novel observation structure to systematically model insertion attacks within PN frameworks for DESs and generate an ERG incorporating this structure to identify special markings, including those with negative values, indicative of attacks.
2. We formulate an ILPP to design an observation place for the system under insertion attacks, enabling precise attack identification based on token count and enhancing overall identification efficiency.
3. We verify the proposed approach through practical examples, demonstrating its effectiveness and applicability in real-world scenarios.

The rest of this paper is organized as follows: Section 2 explores the foundational concepts of PNs and the theory of regions, focusing on their application in control place synthesis for the security of DES. Section 3 defines the problem of insertion attacks in DESs. Section 4 elaborates on methodologies for identifying insertion attacks by designing the identification place for the system under these types of attacks. This section explains the model with observations, and its ERG then the formulation of an ILPP to design an identification place. Section 5 provides practical examples to verify the proposed approach. Section 6, presents a comparative analysis of the proposed method for insertion attack identification in DESs using PNs with an observer, contrasting it with existing approaches in the literature. Finally, Section 7 summarizes the key contributions and explores future research directions to enhance the security and resilience of DES.

## 2 Preliminaries

This section explores the foundational concepts of PNs and the theory of regions, with a specific focus on their application in control place synthesis to enhance the security of DES. PNs are a distinct type of structured network characterized by their two unique node types: places and transitions [53]. A PN can be mathematically represented as a four-tuple N=(P,T,F,W), where *P* represents the set of places, *T* represents the set of transitions, which are finite and mutually exclusive. Visually, places are represented by circles, while transitions are illustrated using bars. The flow relation between these entities, denoted as the set *F* ⊆ (*P* × *T*) ∪ (*T* × *P*), is characterized by directed arcs. The function W:(P×T)∪(T×P)→N={0,1,2,…} defines a weight allocation from (*P* × *T*) ∪ (*T* × *P*) to N={0,1,2,…}. For each arc in *F*, it is assigned a positive weight; otherwise, its weight remains zero, as described by *W*(*x*, *y*) > 0 if (*x*, *y*) ∈ *F* and *W*(*x*, *y*) = 0 otherwise, with *x*, *y* ∈ *P* ∪ *T*. A state or marking, represented as *M*, is defined by the vector $M={\left[M\left(p_{1}\right),M\left(p_{2}\right),\ldots ,M\left(p_{\left|P\right|}\right)\right]}^{T}\in N^{\left|P\right|}$, where *M*(*p*_*i*_) specifies the tokens number in place *p*_*i*_, (*i* ∈ {1, 2, …, |*P*|}) at *M* as particular marking.

A net is pure if for all (*x*, *y*) ∈ (*P* × *T*) ∪ (*T* × *P*), *W*(*x*, *y*) > 0 indicates *W*(*y*, *x*) = 0. For a pure net *N*, its incidence matrix [*N*] has dimensions |*P*| × |*T*| and is an integer matrix characterized by [*N*](*p*, *t*) = *W*(*t*, *p*) − *W*(*p*, *t*). A transition, *t* ∈ *T*, becomes enabled at *M*, which is denoted as *M*[*t*〉 if *M*(*p*) ≥ *W*(*p*, *t*), for all *p* ∈ ^•^ *t*, where ^•^ *t* = {*p*∣(*p*, *t*)∈*F*} is the preset of *t*. If a transition is enabled at a marking *M*, it can be fired, resulting in a new marking *M*′. We use *M*[*t*〉*M*′ to indicate that by firing *t* a marking *M*′ is reached from *M*, such that *M*′(*p*) = *M*(*p*) − *W*(*p*, *t*) + *W*(*t*, *p*), for all *p* ∈ *P*. Given transitions sequence, *α* = *t*_1_ *t*_2_…*t*_*k*_ ∈ T*, *α* is enabled at *M*, written as *M*[*α*〉, if there are intermediary markings *M*_1_, *M*_2_, …, *M*_*k*−1_ satisfying *M*[*t*_1_〉*M*_1_[*t*_2_〉*M*_2_…*M*_*k*−1_[*t*_*k*_〉. Using *M*[*α*〉*M*_*k*_, we express that *M*_*k*_ is reached by firing sequence *α* from *M*. The initial state of a PN is symbolized as *M*_0_, and $\left(N,M_{0}\right)$ forms a system initialized at *M*_0_. This system’s language is characterized by $L\left(N,M_{0}\right)=\left\{\alpha \in T^{*}|M_{0}[\alpha \rangle \right\}$, and its reachability set is $R\left(N,M_{0}\right)=\left\{M|\exists \alpha \in T^{*},M_{0}[\alpha \rangle M\right\}$. If a PN system $\left(N,M_{0}\right)$ possesses a limit $B\in N^{+}=\left\{1,2,\ldots \right\}$ such that *M*(*p*) ≤ *B*, for all *p* ∈ *P*, for all $M\in R(N,M_{0})$, it is termed bounded. Otherwise, it is unbounded.

A transition *t* is said to be live at a marking *M* if for all $M^{\prime }\in R\left(N,M\right)$, there exists $M^{\prime \prime }\in R\left(N,M^{\prime }\right)$, such that *M*″[*t*〉. In contrast, a dead transition *t* at marking *M* means that for all $M^{\prime }\in R\left(N,M\right),t$ is disabled at *M*′. A net system $\left(N,M_{0}\right)$ is live if, for all *t* ∈ *T*, *t* is live at *M*_0_. Furthermore, the RG of $\left(N,M_{0}\right)$ [12] is defined as a directed graph that represents the state space of a PN. The RG is defined as a two-tuple $\left(N,M_{0}\right)=\left(V,E\right)$, where *V* is a set of nodes, such that each node represents a unique marking *M* of the PN, and *E* ⊆ *V* × *V* is a set of directed edges. An edge from node *M* to node *M*′ labeled by transition *t* exists if and only if *M*′ is reachable from *M* by firing *t*. Importantly, these directed arcs indicate transition firings that facilitate a shift from one marking to another.

The theory of regions for control place synthesis, formulated by Ghaffari et al. [54–56], represents an advanced method for improving system control and supervision in the field of PN. This method emphasizes the strategic incorporation of control places into the original PN model, with the goal of more effectively regulating the system’s behavior. The integration of a control place, denoted as *p*_*s*_, alongside its initial markings *M*_0_(*p*_*s*_) and associated incidence vectors $\left[N\right]\left(p_{s},.\right)$, is grounded in a linear algebraic framework that addresses three primary scenarios: reachability conditions, marking/transition separation instance equivalences, and fundamental cycle principles. The reachability condition ensures that, from the system’s initial state, all legal markings are reachable. Let *R*(*N*, *M*_0_) represent the markings set in the PN. Legal markings whose set is denoted as $M_{L}\subseteq R\left(N,M_{0}\right)$ are those that satisfy the system constraints. Illegal markings, denoted as $M_{\bar{L}}$, are the ones that violate certain conditions. For any legal marking, it should not be forbidden by *p*_*s*_. Hence, we have

$$\begin{array}{c} M_{0}\left(p_{s}\right)+\left[N\right]\left(p_{s},\cdot \right){\vec{\Gamma }}_{M_{i}}\geq \text{0}\;,\forall M_{i}\in M_{L} \end{array}$$ (1)

A path in the RG from *M*_0_ to a marking *M*_*i*_ signifies a succession of transitions that, when executed in sequence, leads the system from its initial marking to *M*_*i*_. $\Gamma_{M_{i}}$ represents any non-directed path from *M*_0_ to *M*_*i*_. ${\vec{\Gamma }}_{M_{i}}$ is a T-vector, and ${\vec{\Gamma }}_{M_{i}}\left(t\right)$ represents the algebraic sum of all instances of *t* in $\Gamma_{M_{i}}$. Furthermore, each PN monitor must be capable of resolving at least one banned event (*M*_*i*_, *t*), where the firing of *t* at *M*_*i*_ results in an illegal marking. Eq (2) is called the event separation condition of (*M*_*i*_, *t*). The set of all possible pairs (*M*_*i*_, *t*), where *M*_*i*_ is a reachable marking and *t* is enabled at *M*_*i*_, is called the set of marking/transition separation instance (MTSI) if the firing of *t* at *M*_*i*_ results in an illegal marking.

$$\begin{array}{c} M_{0}\left(p_{s}\right)+\left[N\right]\left(p_{s},\cdot \right){\vec{\Gamma }}_{M_{i}}+\left[N\right]\left(p_{s},t\right)\leq -1 \end{array}$$ (2)

The cycle conditions are crucial for maintaining system invariance and ensuring that the system can maintain its cyclic behavior. The control place *p*_*s*_ must fulfill the cycle equations that are captured by:

$$\begin{array}{c} \left[N\right]\left(p_{s},\cdot \right)\cdot \vec{\sigma }=0,\forall \sigma \in \Delta \end{array}$$ (3)

where the cycles in the RG are sequences of directed edges (transitions) that start and end at the same node (marking), indicating that the PN can return to a previous state after executing a sequence of transitions, Δ represents the set cycles exist in the RG, and $\vec{\sigma }$ is the vector of the algebraic sum of instances of *t* in *σ*. These equations ensure that the introduction of control places does not disrupt the system’s inherent cyclic properties. By addressing these three scenarios through a linear system of equations, the theory enables the calculation of a PN supervisor consisting of control places that collectively enforce the desired system behavior.

## 3 Problem statement

The study addresses the challenge of identifying attacks in DESs that are modeled by PNs. Specifically, it focuses on insertion attacks as one type of sensor attack. These attacks can lead to incorrect decisions by the observers. In PN-based DES models, sensor arrays collect observational data through transition sequences. However, these systems face vulnerabilities to sensor attacks. These attacks can significantly impair the systems’ functionality, performance, and safety, deviating them from their predefined operational specifications. The critical issue occurs when attackers manipulate these sequences by inserting transitions to disrupt the system or inflict damage covertly. To address this issue, the study introduces an observer component within the PN model to monitor event sequences. The research emphasizes the importance of addressing these control challenges to maintain the integrity and reliability of DESs under adversarial conditions.

The insertion attacks are represented as unauthorized modifications to the PN, which involve adding transitions. An insertion attack can be defined as a set ${\bar{T}}_{\text{in}}\subseteq T$, where *T* represents the complete set of transitions within the PN. The set ${\bar{T}}_{\text{in}}$ represents the transitions that the attacker is capable of inserting into the system. In other words, it is a set of transitions that could be falsely reported as having fired. An insert attack on a PN model is modeled within the framework of a PN structure $\left(N,M_{0}\right)$, with N=(P,T,F,W). The attack specifically targets transitions, introducing a set of attack-specific transitions ${\bar{T}}_{\text{in}}$ to the original transition set *T*, resulting in an augmented set of transitions $T_{\text{attack}}=T\cup {\bar{T}}_{\text{in}}$. This modification reflects the insertion attack, thereby altering the flow relations within the PN model to $F_{\text{attack}}=\bar{F}\cup {\bar{F}}_{\text{in}}$, which incorporate connections between these new transitions and specific places *P*_*o*_, modeling the attack’s impact. The attacked model, represented as $\left(N^{\prime \prime },M_{0}^{\prime }\right)$ with $N^{\prime \prime }=\left(\bar{P},T_{\text{attack}},F_{\text{attack}},\bar{W}\right)$, encapsulates the dynamics under insertion attacks, facilitating an analysis of how such attacks can modify the standard operational flows of the system.

The formulation problem started with a given PN system $\left(N,M_{0}\right)$ vulnerable to insertion attack with its observation $\left(N^{\prime },M_{0}^{\prime }\right)$, with $N^{\prime }=\left(\bar{P},T,\bar{F},\bar{W}\right)$, and a system $\left(N^{\prime \prime },M_{0}^{\prime }\right)$ under an insertion attack with $N^{\prime \prime }=(\bar{P},T_{\text{attack}\;},F_{\text{attack}\;},\bar{W}$). The solution to this challenge starts by defining and generating the ERG with observations. Then, an ILPP is formulated to design an identification place. Finally, we can easily identify attacks by observing the number of tokens in a specially designed identification place, which provides a direct and effective identification technique.

## 4 Proposed method

This section outlines the methodology for designing the identification place within a system subject to insertion attacks. The identification place is a strategically designed component within the PN model of a DES, serving as a key element in detecting and responding to these types of insertion attacks. The design process involves a thorough analysis of the system’s operational dynamics to pinpoint where and how these illicit insertions could occur. This entails mapping out the token flow within the PN and identifying potential weaknesses that an attacker could exploit. The identification place is then positioned within the PN to monitor these vulnerable points effectively. This part aims to design an identification place for the system under insertion attack. The method takes three essential steps to address this issue of attack identification. Here are the details of the three steps:

### 4.1 The system model with observations under insertion attacks

This section discusses the structure of observations and the methodology for net synthesis, integrating the original PN with the observations. The primary focus is on delineating the role and dynamics of observation places, which are critical for understanding the system’s behavior in the presence of insertion attack-induced event sequence changes. An original PN system model, denoted as $\left(N,M_{0}\right)$ with N=(P,T,F,W), is extended with an observation structure *OB* = (*P*_*o*_, *F*_*o*_). The observation structure is designed to mirror the original net system state space by introducing a corresponding finite set of observation states *P*_*o*_, such that for every place *p*_*i*_ in the original net N, there exists a corresponding observation state $p_{i}^{\prime }$ in *P*_*o*_. Flow relations *F*_*o*_ are adapted from N, ensuring corresponding flows between observation places and transitions as shown in Definition 1.

**Definition 1**: For a PN system model $\left(N,M_{0}\right)$ with N=(P,T,F,W), the observation structure of $\left(N,M_{0}\right)$ is defined as *OB* = (*P*_*o*_, *F*_*o*_), where:

- *P*_*o*_ is a finite set of corresponding places such that for all *p*_*i*_ ∈ *P* in N, then $p_{i}^{\prime }\in P_{o}$.
- *F*_*o*_ ⊆ (*P*_*o*_ × *T*) ∪ (*T* × *P*_*o*_), where $\left(p_{j}^{\prime },t_{i}\right)\in F_{o}$ if (*p*_*j*_, *t*_*i*_) ∈ *F* and $\left(t_{i},p_{j}^{\prime }\right)\in F_{o}$ if $(t_{i},p_{j}^{\prime })\in F.$

The combined PN model $O(N^{\prime },M_{0}^{\prime })$ integrates an original PN system $\left(N,M_{0}\right)$ with an observation structure *OB* = (*P*_*o*_, *F*_*o*_). The observation structure contributes additional observation places *P*_*o*_ and flow relations *F*_*o*_ to the original model. The resulting combined system $N^{\prime }$ is defined by a unified set of places $\bar{P}=P\cup P_{o}$, an aggregated set of flow relations $\bar{F}=F\cup F_{o}$, and the extended initial marking $M_{0}^{\prime }=(M_{0}\left(p_{i}\right)+M_{0}\left(p_{i}^{\prime }\right)$ as shown in Definition 2.

**Definition 2**: For a PN system model $\left(N,M_{0}\right)$ with N=(P,T,F,W) and its observation structure *OB* = (*P*_*o*_, *F*_*o*_), the combined system (combining the original net with its observation structure) is represented as $O(N^{\prime },M_{0}^{\prime })$ with $N^{\prime }=(\bar{P}$, $T,\bar{F},\bar{W})$ where:

-
$\bar{P}=\left(P\cup P_{o}\right)$ ;
-
$\bar{F}=\left(F\cup F_{o}\right)$ ;
- The function $\bar{W}$ is defined as: $\bar{W}:\left(\bar{P}\times T\right)\cup \left(T\times \bar{P}\right)\to N=\left\{0,1,2,\ldots \right\}$ with the operational rule $\bar{W}\left(x,y\right)>0$ if $\left(x,y\right)\in \bar{F}$ and $\bar{W}\left(x,y\right)=0$ otherwise, for all $x,y\in \bar{P}\cup T$;
-
$M_{0}^{\prime }=\left(M_{0}\left(p_{i}\right)+M_{0}\left(p_{i}^{\prime }\right)\right)$ .

An insert attack occurs in the PN model $\left(N,M_{0}\right)$ with N=(P,T,F,W) at a certain transition *t*_*i*_ ∈ *T*. The model has an observation system $\left(N^{\prime },M_{0}^{\prime }\right)$ and $N^{\prime }=\left(\bar{P},T,\bar{F},\bar{W}\right)$.

**Definition 3**: For a PN model with observations $N^{\prime }=(\bar{P},T,\bar{F},\bar{W})$ under an insertion attack *T*_*in*_, its attacked net system $\left(N^{\prime \prime },M_{0}^{\prime }\right)$ with $N^{\prime \prime }=\left(\bar{P},T_{\text{attack}\;},F_{\text{attack}\;},\bar{W}\right)$ is defined as follows:

-
$T_{\text{attack}\;}=T\cup {\bar{T}}_{\text{in}\;}$ where ${\bar{T}}_{\text{in}}$ is the set of the corresponding transitions of *T*_in_ so that $t_{i}^{+}\in {\bar{T}}_{in}$ if *t*_*i*_ ∈ *T*_*in*_.
-
$F_{\text{attack}\;}=\bar{F}\cup {\bar{F}}_{\text{in}\;}$ and ${\bar{F}}_{\text{in}\;}\subseteq \left({\bar{T}}_{\text{in}\;}\times P_{o}\right)\cup \left(P_{o}\times {\bar{T}}_{\text{in}\;}\right)$ where $\left(p_{j}^{\prime },t_{i}\right)\in F_{o}$ if (*p*_*j*_, *t*_*i*_)∈*F* and $\left(t_{i},p_{j}^{\prime }\right)\in F_{o}$ if $(t_{i},p_{j}^{\prime })\in F.$

Definition 3 presents a PN model, denoted as $\left(N^{\prime \prime },M_{0}^{\prime }\right)$, to analyze the dynamics under insertion attacks. This model integrates additional transitions, ${\bar{T}}_{\text{in}}$, with the original set *T*, to encapsulate the attack vectors. These augmented transitions, alongside the original ones, form the set $T_{\text{attack}}=T\cup {\bar{T}}_{\text{in}}$, where ${\bar{T}}_{\text{in}}$ corresponds to a subset of *T* directly related to insertion attacks. The flow relations, $F_{\text{attack}}=\bar{F}\cup {\bar{F}}_{\text{in}}$, expand to include these new transitions, linking them to specific places *P*_*o*_, thus modeling the attack’s impact on the system. This structure facilitates an in-depth exploration of how insertion attacks alter the system’s standard operational flows, offering insights into the vulnerabilities within PN models. Within the observation model, the observation places *P*_*o*_ cannot distinguish between the transition *t*_*i*_ and its corresponding insertion transition $t_{i}^{+}$. This indistinguishability arises because both types of transitions when activated produce similar or identical effects with respect to the observable changes in the system’s state as recorded by *P*_*o*_.

### 4.2 The extended reachability graph for the system model with observations under insertion attacks

**Fig 2:**
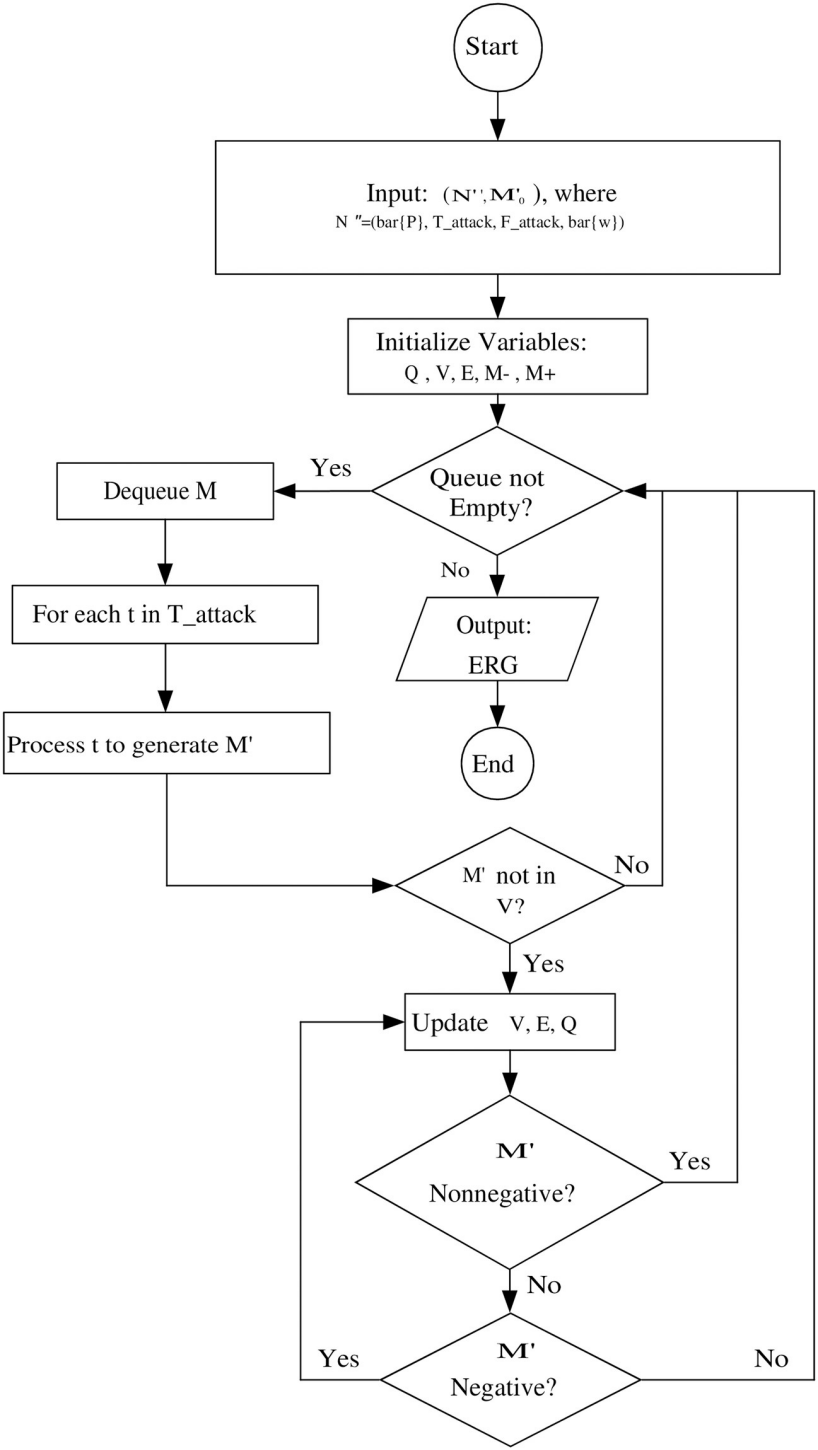
The flowchart of the ERG generation for $\left(N^{\prime \prime },M_{0}^{\prime }\right)$.

This part presents an ERG for $\left(N^{\prime \prime },M_{0}^{\prime }\right)$ and determines a set of markings that is divided into a set of negative markings $M^{-}$ and a set of non-negative markings $M^{+}$. The negative markings indicate the occurrence of an attack in this state, and the output transition firing sequence stops. For a PN system under attack with its observation $N^{\prime \prime }$, the places in *P* represent the state of the original system but those in *P*_*o*_ show the state of observations. By considering that *P*_*o*_ is used to only observe the behavior of the original system, it should not disable any transition. In this sense, the disable and enable condition of a transition is decided only by the places in *P*. Hence, we need to extend the meaning of tokens in *P*_*o*_ whose number is allowed to be negative. This extended concept is useful to identify the occurrences of attacks. The flowchart in Fig 2 illustrates the process of generating an ERG for a PN model under an insertion attack. It includes steps for initializing variables, processing transitions, and updating the graph structure based on non-negative and negative markings.

**Definition 4**: For a PN model with observations under an insertion attack $\left(N^{\prime \prime },M_{0}^{\prime }\right)$ with $N^{\prime \prime }=\left(\bar{P}=\left(P\cup P_{o}\right),T_{\text{attack}\;}=\left(T\cup {\bar{T}}_{\text{in}}\right),F_{\text{attack}\;}=\left(\bar{F}\cup {\bar{F}}_{\text{in}}\right),\bar{W}\right)$, a given transition *t* ∈ *T*_attack_ is enabled if the following condition holds:

- If *t* ∈ *T*, for all *p*_*i*_ ∈ ^•^ *t*, *M*(*p*_*i*_) ≥ *W*(*p*_*i*_, *t*).
- If $t\in {\bar{T}}_{\text{in}}$, *t* is always enabled. [27, 41, 51]

**Algorithm 1** Generation of the ERG for a system with its observation under an insertion attack

1: **Input**: A system PN model with observation under an insertion attack $\left(N^{\prime \prime },M_{0}^{\prime }\right)$, where $N^{\prime \prime }=(\bar{P}=\left(P\cup P_{o}\right),T_{\text{attack}\;}=\left(T\cup {\bar{T}}_{\text{in}\;}\right),$ and $F_{\text{attack}\;}=\left(\bar{F}\cup {\bar{F}}_{\text{in}\;}\right),\bar{w})$

2: **Output**: $ERG≔(V,E,\left\{M^{-}\right\},\left\{M^{+}\right\})$

3: $Q≔\{M_{0}^{\prime }\}$

4: $V≔\{M_{0}^{\prime }\}$

5: *E* ≔ ∅

6: $M^{-}≔\emptyset$

7: $M^{+}≔\emptyset$

8: **while** *Q* ≠ ∅ **do**

9:  *M* ≔ dequeue (*Q*)

10:  **for** each *t* ∈ *T*_attack_ **do**

11:   **if** *t* ∈ *T* **then**

12:    Generate a new marking *M*′ ≔ *M* by firing *t* represented by $M\underset{t}{\to }M^{\prime }$

13:    **if** *M*′ ∉ *V* **then**

14:     *V* ≔ *V* ∪ {*M*′}

15:     enqueue (*Q*, *M*′)

16:     *E* ≔ *E* ∪ {(*M*, *M*′)}

17:     $M^{+}≔M^{+}\cup \left\{M^{\prime }\right\}$

18:    **end if**

19:   **end if**

20:   **if** $t\in {\bar{T}}_{\text{in}}$ **then**

21:    Generate a new marking *M*′ ≔ *M* by firing *t* represented by $M\underset{t}{\to }M^{\prime }$

22:    **if** *M*′ ∉ *V* **then**

23:     *V* ≔ *V* ∪ {*M*′}

24:     *E* ≔ *E* ∪ {(*M*, *M*′)}

25:     **if** *M*′ is a nonnegative marking **then**

26:      enqueue (*Q*, *M*′)

27:      $M^{+}≔M^{+}\cup \left\{M^{\prime }\right\}$

28:      **if** *M*′ is a negative markings **then**

29:       $M^{-}=M^{-}\cup \left\{M^{\prime }\right\}$

30:      **end if**

31:     **end if**

32:    **end if**

33:   **end for**

35:  **end while**

36: **Output** *ERG*.

37: **End**.

In the PN system model $\left(N^{\prime \prime },M_{0}^{\prime }\right)$, when a transition *t* fires, it results in the generation of a new marking *M*′ from the current marking *M*. The resulting marking *M*′ can be categorized as either negative or nonnegative, depending on the token distribution within the observation places *P*_*o*_. Specifically, a marking *M*′ is deemed negative if at least one instance exists observation place $p_{i}^{\prime }\in P_{o}$ with a token count less than zero, denoted by $M^{\prime }\left(p_{i}^{\prime }\right)<0$. Conversely, a marking *M*′ is considered to be nonnegative for every place $p\in \bar{P}$ in the system (including both the standard places in *P* and the observation places in *P*_*o*_), the token count in place *p* at marking *M*′ satisfies the condition *M*′(*p*) ≥ 0.

**Definition 5**: Consider the PN model with observations under an insertion attack $\left(N^{\prime \prime },M_{0}^{\prime }\right)$ with $N^{\prime \prime }=\left(\bar{P}=\left(P\cup P_{o}\right),T_{\text{attack}\;}=\left(T\cup {\bar{T}}_{\text{in}}\right),F_{\text{attack}\;}=\left(\bar{F}\cup {\bar{F}}_{\text{in}}\right),\bar{W}\right)$ and an initial marking $M_{0}^{\prime }$, an $ERG=(V,E,M^{-},M^{+})$ is defined as follows:

- *V* is a set of nodes, where every node signifies a unique marking *M* of the PN.
- *E* ⊆ *V* × *V* is a set of directed edges. An edge from node *M* to node *M*′ labelled by transition *t* exists if and only if *M*′ is reachable from *M* by firing *t*.
-
$M^{-}$ is a set of negative markings.
-
$M^{+}$ is a set of non-negative markings.

**Theorem 1**: In a PN model with observations under an attack scenario $\left(N^{\prime \prime },M_{0}^{\prime }\right)$, a negative marking $M^{\prime }\in M^{-}$ indicates the occurrence of an insertion attack.

*Proof*: Let $\left(N^{\prime \prime },M_{0}^{\prime }\right)$ be the PN model with observations under an attack, where $N^{\prime \prime }=(\bar{P}=\left(P\cup P_{o}\right),T_{\text{attack}\;}=\left(T\cup {\bar{T}}_{\text{attack}\;}\right),$ and $F_{\text{attack}\;}=\left(\bar{F}\cup {\bar{F}}_{\text{attack}\;}\right),\bar{W})$. Suppose that *M*′ is a marking of the PN model $N^{\prime \prime }$ such that there exists at least one place $p_{i}^{\prime }\in P_{o}$ with a token count less than zero, i.e., $M^{\prime }\left(p_{i}^{\prime }\right)<0$. Since the places in *P*_*o*_ represent the observed states of the system, a negative token count in any $p_{i}^{\prime }\in P_{o}$ indicates an unexpected or unauthorized state of the system. This deviation from the expected behavior signifies an anomaly or attack on the system. Therefore, the presence of a negative marking *M*′ in the PN model $\left(N^{\prime \prime },M_{0}^{\prime }\right)$ conclusively demonstrates the occurrence of an attack, as it deviates from the normal operational state represented by non-negative markings.

### 4.3 Formulate the ILPP to design the identification place for the system under insertion attacks

This section explains establishing an identification place to identify potential attacks through a well-defined ILPP. The main goal is to correctly identify an attack by using the identification rule. Let *p*_*s*_ be an identification place to identify an insertion attack. Our identification rule of an insertion attack is that an attack is present if *M*(*p*_*s*_) < *b*, where *M*(*p*_*s*_) is a measurable attribute associated with a position identifier *p*_*s*_, and *b* is the limit for normal activity. To achieve this, it is imperative to articulate the design of the ILPP with precision, which entails defining the variables, constraints, and objective functions that will guide the optimization process. The key steps to formulate the ILPP are explained as follows:

**Step 1: Variables** Initially, variables are defined for an ILPP to model critical aspects of an identification place, designated as *p*_*s*_. For each connection from *p* to *t*, a variable *x*_*p*,*t*_ represents the tokens consumed by transition *t* from place *p*. Specifically, for the identification place *p*_*s*_, the variable *M*_0_(*p*_*s*_) indicates its initial token count. The incidence vector $\left[N_{p_{s}}\right]$ is introduced, where $\left[N_{p_{s}}\right]\left(t\right)=W\left(t,p_{s}\right)-W\left(p_{s},t\right)$ for all *t* ∈ *T*_*attack*_, simplifying the representation of the net token change as *x*_*p*_*s*_, *t*_ = $\left[N_{p_{s}}\right]\left(t\right)$ for transitions involved in attacks.
**Step 2: Constraints** Following the variable definition, the construction of the ILPP necessitates the formulation of constraints. These constraints are developed to ensure the operational efficacy of the observation place while adhering to the identification rule. Specifically, they encompass conditions that enforce the detection capabilities within the specified bounds, the allocation limits of available resources, and any other operational parameters critical to the place’s functionality. The constraints are meticulously crafted to embody the practical and theoretical aspects that govern the observation place’s design and operational dynamics. The constraints of ILP are mentioned as follows:
  1. *Reachability Condition Constraint*: This constraint ensures that for each nonnegative marking, the sequence of transitions from the initial state *M*_0_ is reachable. Let M represent the set of reachable markings in the PN. Let *M*_*i*_ be a nonnegative marking, i.e., $M_{i}\in M^{+}$. A path in the ERG from an initial marking *M*_0_ to a marking *M*_*i*_ represents a sequence of transitions that, when fired sequentially, leads the system from its initial marking to *M*_*i*_. The vector ${\vec{\Gamma }}_{M_{i}}$ that corresponds to the path from the *M*_0_ to the *M*_*i*_ represents a sequence of transitions or events leading from the initial marking to *M*_*i*_. The set of observation places *P*_*o*_ cannot distinguish between the transition *t*_*i*_ and its corresponding insertion transition $t_{i}^{+}$. This indistinguishability arises because both types of transitions when activated produce similar or identical effects in terms of the observable changes in the system’s state as recorded by *P*_*o*_. It means that a transition *t*_*i*_ and its insertion transition $t_{i}^{+}$ are considered the same in the observation places. By our identification rule, the number of tokens in the identification place at a nonnegative marking must be greater than or equal to a constant *b*. Hence, we have
    $$\begin{array}{c} M_{0}\left(p_{s}\right)+\left[N\right]\left(p_{s},\cdot \right){\vec{\Gamma }}_{M_{i}}\geq b,\forall M_{i}\in M^{+} \end{array}$$ (4)
  2. *Negative Impact Pair (NIP) Conditions Constraint*: Each PN monitor must be capable of identifying at least one critical event, (*M*_*k*_, *t*), where the firing of *t* at *M*_*k*_ leads to a negative marking or undesirable state. This condition, referred to as the Negative Impact Condition for a pair (*M*_*k*_, *t*), signifies scenarios where the system transitions into a prohibited or harmful state due to the firing of some insertion transition in the path from *M*_0_ to marking *M*_*k*_. The collection of all such pairs (*M*_*k*_, *t*), where *M*_*k*_ is a reachable marking leading to a negative outcome upon the firing of *t*, is termed the set of NIPs denoted as Ω. In this case, we should ensure that the count of tokens in the identification place is less than the constant *b*. This is mathematically expressed as:
    $$\begin{array}{c} M_{0}\left(p_{s}\right)+\left[N\right]\left(p_{s},\cdot \right){\vec{\Gamma }}_{M_{k}}+\left[N\right]\left(p_{s},t\right)\leq b-1,\forall \left(M_{k},t\right)\in \Omega \end{array}$$ (5)
  3. *Cycle Conditions Constraint*: The cycle conditions are crucial for maintaining system invariance and ensuring the system’s ability to revert to its initial state or maintain its cyclic behavior without violating any constraints. A cycle in the ERG is a sequence of directed edges (transitions) that start and end at the same node (marking), indicating that the PN can return to a previous state after executing a sequence of transitions. Δ denotes the set of existing cycles in the ERG, and $\vec{\sigma }$ is the vector of the algebraic sum of *t* occurrences in *σ*. The identification place *p*_*s*_ must fulfill the cycle equations that are captured by:
    $$\begin{array}{c} \left[N\right]\left(p_{s},\cdot \right)\cdot \vec{\sigma }=0,\forall \sigma \in \Delta \end{array}$$ (6)
    These equations guarantee that the integration of identification places preserves the system’s intrinsic cyclic properties.
**Step 3: Objective function** The objective function is contingent upon the goal of designing the identification place to identify an attack. For a standard form of ILPP, we add an objective function such as minimizing (*M*_0_(*p*_*s*_) or *b*) or maximizing (*M*_0_(*p*_*s*_) or *b*). Therefore, the subsequent ILPP is formulated to design an identification place *p*_*s*_, which is referred to as the minimal initial marking of the identification place problem (MIP).

MIP:

$$\text{min}\;M_{0}\left(p_{s}\right)$$

subject to

$$\begin{array}{cc} & M_{0}\left(p_{s}\right)+\left[N\right]\left(p_{s},\cdot \right){\vec{\Gamma }}_{M_{i}}\geq b,\forall M_{i}\in M^{+}\\ & M_{0}\left(p_{s}\right)+\left[N\right]\left(p_{s},\cdot \right){\vec{\Gamma }}_{M_{k}}+\left[N\right]\left(p_{s},t\right)\leq b-1,\forall \left(M_{k},t\right)\in \Omega \\ & \left[N\right]\left(p_{s},\cdot \right)\cdot \vec{\sigma }=0,\forall \sigma \in \Delta \\ & M_{0}\left(p_{s}\right),b\in \left\{0,1,2,\ldots \right\},\left[N\right]\left(p_{s};t\right)\in \left\{\ldots ,-2,-1,0,1,2,\ldots \right\} \end{array}$$

The linear system comprises reachability situations, Negative Impact Pair (NIP) Conditions equivalences, and the fundamental cycle principles. The linear system solution results in an identification place *p*_*s*_. Then, the place is utilized to identify an attack by checking the tokens in *p*_*s*_ if *M*(*p*_*s*_) < *b*. In this case, the attack is detected and gets an identified PN of the system $\left(N^{s},M_{0}^{s}\right)$.

**Algorithm 2** Computation of identification place for the system under insertion attacks

1: **Input**: PN model $\left(N,M_{0}\right)$ with N=(P,T,F,W) and a PN observation model with attacks $\left(N^{\prime \prime },M_{0}^{\prime }\right)$ with $N^{\prime \prime }=\left(\bar{P},T_{\text{attack}\;}=T\cup {\bar{T}}_{\text{in}\;},F_{\text{attack}\;}=\bar{F}\cup {\bar{F}}_{\text{in}\;}\;,\bar{w}\right),$ ERG for $(N^{\prime \prime },M_{0}^{\prime })=\left(V,E,\left\{M^{-}\right\},\left\{M^{+}\right\}\right)$

2: **Output**: An identified controlled PN system $\left(N^{s},M_{0}^{s}\right)$.

3: Compute the set Δ of cycles of ERG and define the vector $\vec{\sigma }$.

4: Formulate MIP and solve it.

5: Let *M*_0_(*p*_*s*_) and $\left[N\right]\left(p_{s},\cdot \right)$ be a solution.

6: Add *p*_*s*_ to $\left(N,M_{0}\right)$ and designate the resulting net system as $\left(N^{s},M_{0}^{s}\right)$.

7: **Output**: $\left(N^{s},M_{0}^{s}\right)$.

8: **End**.

## 5 Practical examples

In this section, we present several practical examples to illustrate the practical implementation and effectiveness of the proposed methods. These examples demonstrate how the theoretical concepts and methodologies discussed earlier can be applied to real-world scenarios, providing a clearer understanding of their utility and impact.

**Fig 3:**
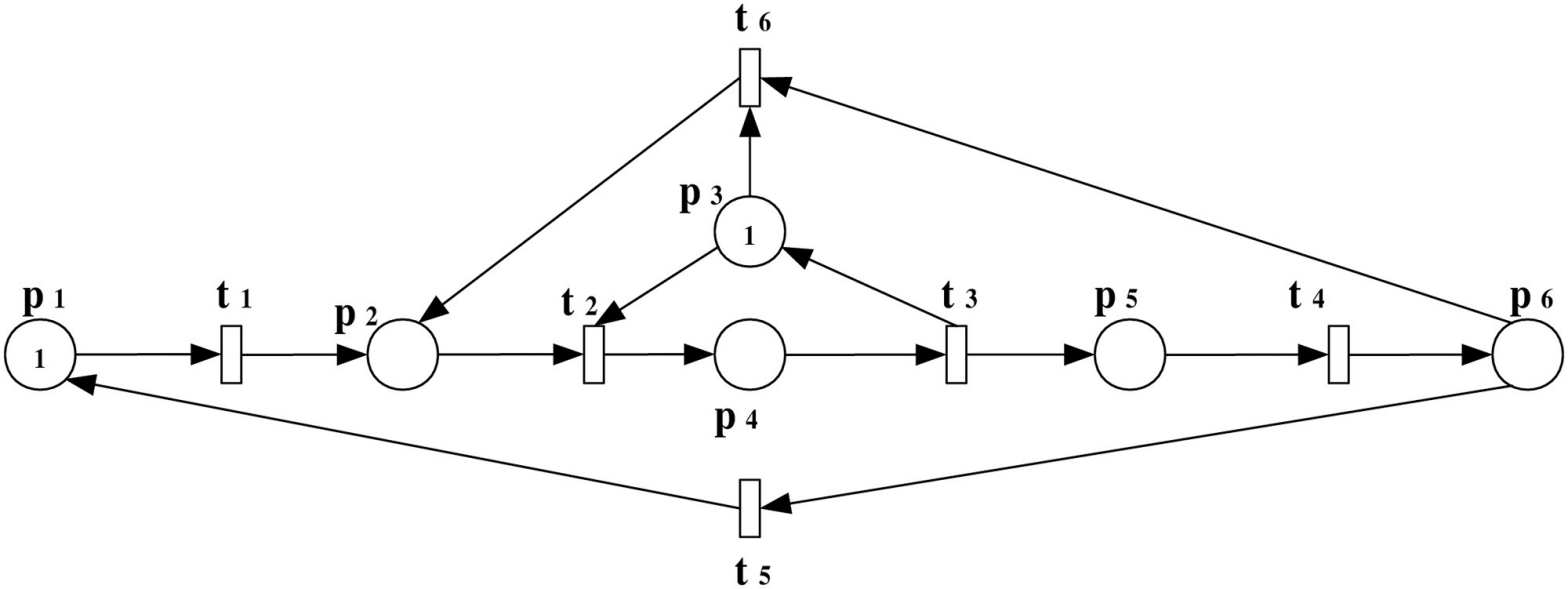
PN model $\left(N,M_{0}\right)$.

**Example 1**: For a bounded PN model, denoted as $\left(N,M_{0}\right)$ with N=(P,T,F,W), as illustrated in Fig 3, we investigate the scenario of an insertion attack at transition *t*_1_. We will study this example step by step as follows:

***Step 1***: *Scenario of an insertion attack and the observation system augmentation*

**Fig 4:**
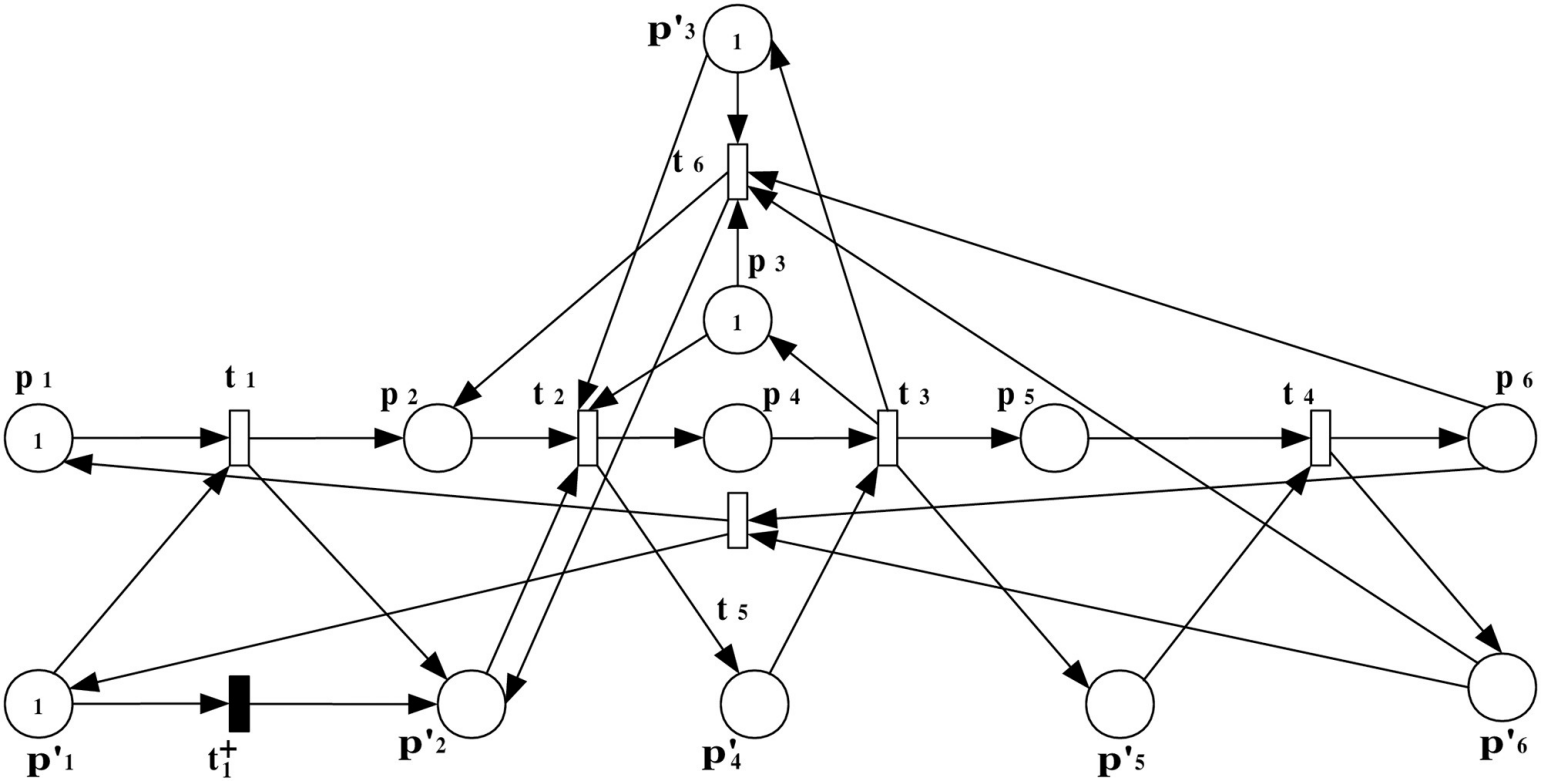
The observation system $\left(N^{\prime \prime },M_{0}^{\prime }\right)$ with an insertion attack transition $t_{1}^{+}$ that occurred at transition *t*_1_.

In this step, a scenario of an insertion attack at a specific transition within the PN model is introduced. The augmented observation system is developed to analyze the impact of the attack on the system’s behavior, highlighting the altered flow and resource allocation. To precisely model and analyze the effects of such an attack within the system, we extend the original PN model to incorporate the attack dynamics. This augmented model, represented as $\left(N^{\prime \prime },M_{0}^{\prime }\right)$ with $N^{\prime \prime }=(\bar{P},T_{\text{attack}}=T\cup \left\{t_{1}^{+}\right\},F_{\text{attack}}=\bar{F}\cup {\bar{F}}_{\text{in}},\bar{W})$, is depicted in Fig 4.

***Step 2***: *ERG generation*

**Fig 5:**
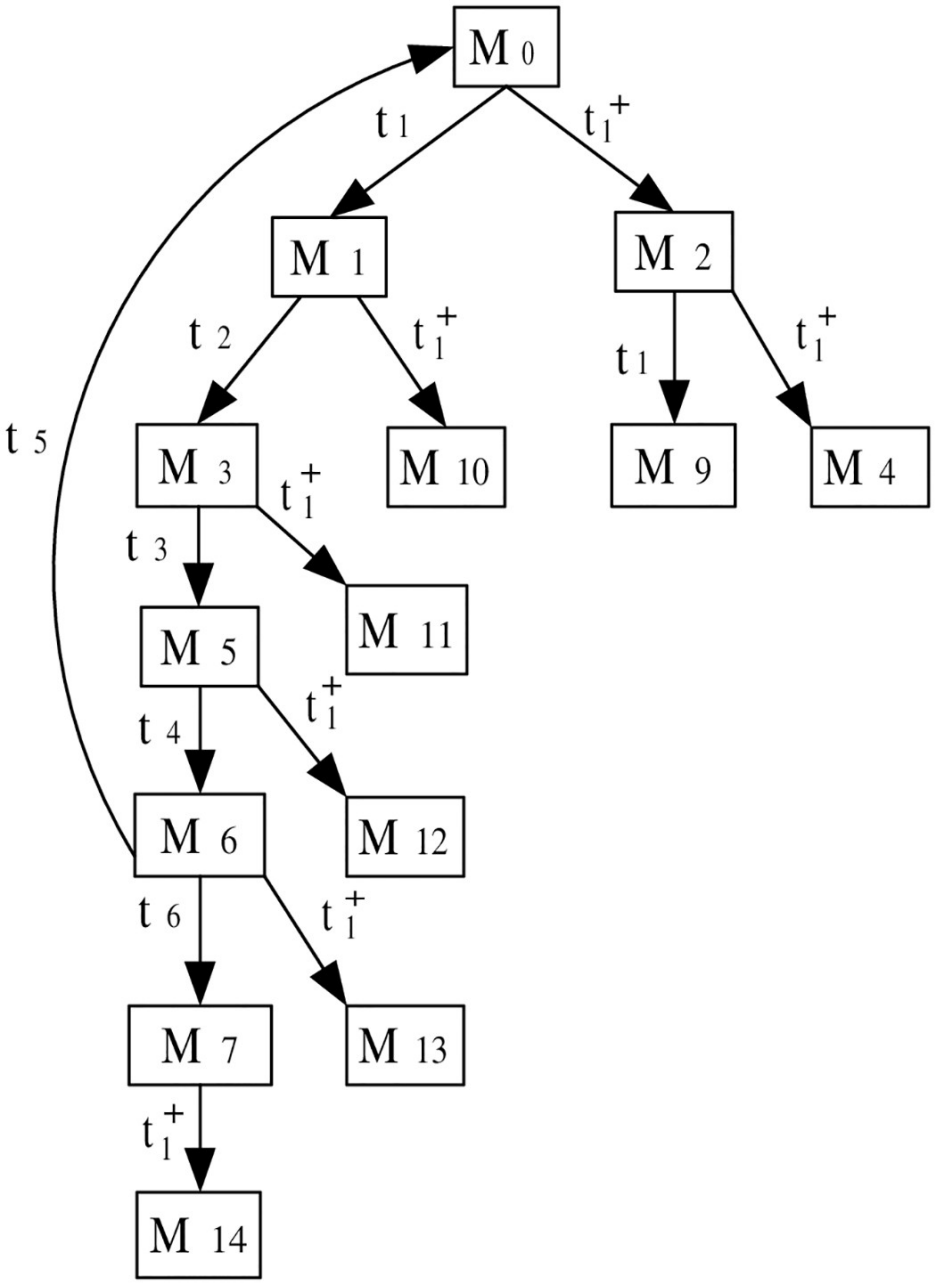
The ERG of $\left(N^{\prime \prime },M_{0}^{\prime }\right)$.

This step explains the generation of the ERG derived from the augmented PN model under attack. The ERG provides a comprehensive view of the system’s states and transitions, illustrating how the attack influences normal operation. The ERG derived from the observation system $\left(N^{\prime \prime },M_{0}^{\prime }\right)$, which provides a comprehensive overview of the system’s state space post-attack, is presented in Fig 5. This ERG serves as a fundamental tool for analyzing the system’s behavior and the implications of the insertion attack on its operational integrity.

The marking (token distribution) of a state *M* can be described as a general expression of a multiset $M=\sum_{p_{i}\in \bar{P}}M\left(p_{i}\right)\cdot p_{i}$, where *M*(*p*_*i*_) represents the token count in place *p*_*i*_, and $\bar{P}$ includes all places in the observation system $\left(N^{\prime \prime },M_{0}^{\prime }\right)$, including both standard places in *P* and observation places in *P*_*o*_ (details shown in S1 File in S1 Data). The markings in Fig 5 are presented as follows:

**Markings**

$$\begin{array}{cc} M_{0} & p_{1}+p_{3}+{p^{\prime }}_{1}+{p^{\prime }}_{3}\\ M_{1} & p_{2}+p_{3}+{p^{\prime }}_{2}+{p^{\prime }}_{3}\\ M_{2} & p_{1}+p_{3}+{p^{\prime }}_{2}+{p^{\prime }}_{3}\\ M_{3} & p_{4}+{p^{\prime }}_{4}\\ M_{4} & p_{1}+p_{3}-{p^{\prime }}_{1}+2{p^{\prime }}_{2}+{p^{\prime }}_{3}\\ M_{5} & p_{3}+p_{5}+{p^{\prime }}_{3}+{p^{\prime }}_{5}\\ M_{6} & p_{3}+p_{6}+{p^{\prime }}_{3}+{p^{\prime }}_{6}\\ M_{7} & p_{2}+{p^{\prime }}_{2}\\ M_{9} & p_{2}+p_{3}-{p^{\prime }}_{1}+2{p^{\prime }}_{2}+{p^{\prime }}_{3}\\ M_{10} & p_{2}+p_{3}-{p^{\prime }}_{1}+2{p^{\prime }}_{2}+{p^{\prime }}_{3}\\ M_{11} & p_{4}-{p^{\prime }}_{1}+{p^{\prime }}_{2}+{p^{\prime }}_{4}\\ M_{12} & p_{3}+p_{5}-{p^{\prime }}_{1}+{p^{\prime }}_{2}+{p^{\prime }}_{3}+{p^{\prime }}_{5}\\ M_{13} & p_{3}+p_{6}-{p^{\prime }}_{1}+{p^{\prime }}_{2}+{p^{\prime }}_{3}+{p^{\prime }}_{6}\\ M_{14} & p_{2}-{p^{\prime }}_{1}+2{p^{\prime }}_{2} \end{array}$$

***Step 3***: *ILPP formulation*

The ILPP is formulated in this step to address the occurrence of negative markings resulting from the attack. In the context of our analysis, negative markings, denoted as *M*_4_, *M*_9_, *M*_10_, *M*_11_, *M*_12_, *M*_13_, and *M*_14_, are indicative of an attack state. The interaction between an identification place *p*_*s*_ and transitions *t*_*i*_ is characterized through a set of variables *x*_*i*_, formalized as $\left[N_{s}\right]\left(p_{s}\right)=\left[x_{1}\;\text{at}\;t_{1}\text{and}\;t_{1}^{+},x_{2}\;\text{at}\;t_{2},x_{3}\;\text{at}\;t_{3}\right]$. For any nonnegative marking *M*, it holds *M*(*p*_*s*_)≥*b*. Hence, we have the following reachability conditions:

$$\begin{array}{c} M_{0}\left(p_{s}\right)\geq b,\\ M_{1}\left(p_{s}\right)=M_{2}\left(p_{s}\right)=M_{0}\left(p_{s}\right)+x_{1}\geq b,\\ M_{3}\left(p_{s}\right)=M_{0}\left(p_{s}\right)+x_{1}+x_{2}\geq b,\\ M_{5}\left(p_{s}\right)=M_{0}\left(p_{s}\right)+x_{1}+x_{2}+x_{3}\geq b,\\ M_{6}\left(p_{s}\right)=M_{0}\left(p_{s}\right)+x_{1}+x_{2}+x_{3}+x_{4}\geq b,\\ M_{7}\left(p_{s}\right)=M_{0}\left(p_{s}\right)+x_{1}+x_{2}+x_{3}+x_{4}+x_{6}\geq b, \end{array}$$

In this example, the cycle in the ERG involves the transitions sequence *t*_1_ → *t*_2_ → *t*_3_, →*t*_4_ → *t*_5_ starting and ending at the same node (starting at the initial marking *M*_0_). The cycle equations are:

$$x_{1}+x_{2}+x_{3}+x_{4}+x_{5}=0,$$

For the negative marking *M*_4_, *M*_9_, *M*_10_, *M*_11_, *M*_12_, *M*_13_, and *M*_14_, it comes:

$$\begin{array}{c} M_{4}\left(p_{s}\right)=M_{9}\left(p_{s}\right)=M_{10}\left(p_{s}\right)=M_{0}\left(p_{s}\right)+2x_{1}\leq b-1,\\ M_{11}\left(p_{s}\right)=M_{0}\left(p_{s}\right)+2x_{1}+x_{2}\leq b-1,\\ M_{12}\left(p_{s}\right)=M_{0}\left(p_{s}\right)+2x_{1}+x_{2}+x_{3}\leq b-1,\\ M_{13}\left(p_{s}\right)=M_{0}\left(p_{s}\right)+2x_{1}+x_{2}+x_{3}+x_{4}\leq b-1,\\ M_{14}\left(p_{s}\right)=M_{0}\left(p_{s}\right)+2x_{1}+x_{2}+x_{3}+x_{4}+x_{6}\leq b-1. \end{array}$$

An objective function is proposed to minimize *M*_0_(*p*_*s*_) in this example. By solving ILPP (details shown in S1 File in S1 Data), an optimal solution with *M*_0_(*p*_*s*_) = 1, *b* = 0, and $\left[N_{p_{s}}\right]=\left[-1,0,0,0,1,0\right]$ is obtained.

***Step 4***: *Verification paths*

Verification paths are examined to ensure that under attack conditions, the system’s transitions lead to safe states. This step focuses on validating the system’s robustness by analyzing the sequences of markings and transitions under normal and compromised conditions. The markings and transitions sequences in Table 1 show that for any marking *M* with *M*(*p*_*s*_) ≤ *b* − 1, there does not exist any path without attack from *M*_0_ to *M* such as *M*_14_, *M*_13_, *M*_12_, *M*_11_, *M*_10_, *M*_9_, and *M*_4_.

**Table 1:** Verification paths.

Markings	Paths $\left(t_{1}t_{1}^{+}t_{2}t_{3}t_{4}t_{5}t_{6}\right)$	Markings	Paths $\left(t_{1}t_{1}^{+}t_{2}t_{3}t_{4}t_{5}t_{6}\right)$
*M*_0_	0000000	*M*_7_	1011101
*M*_1_	1000000	*M*_9_	1100000
*M*_2_	0100000	*M*_10_	1100000
*M*_3_	1010000	*M*_11_	1110000
*M*_4_	0200000	*M*_12_	1111000
*M*_5_	1011000	*M*_13_	1111100
*M*_6_	1011100	*M*_14_	1111101

**Fig 6:**
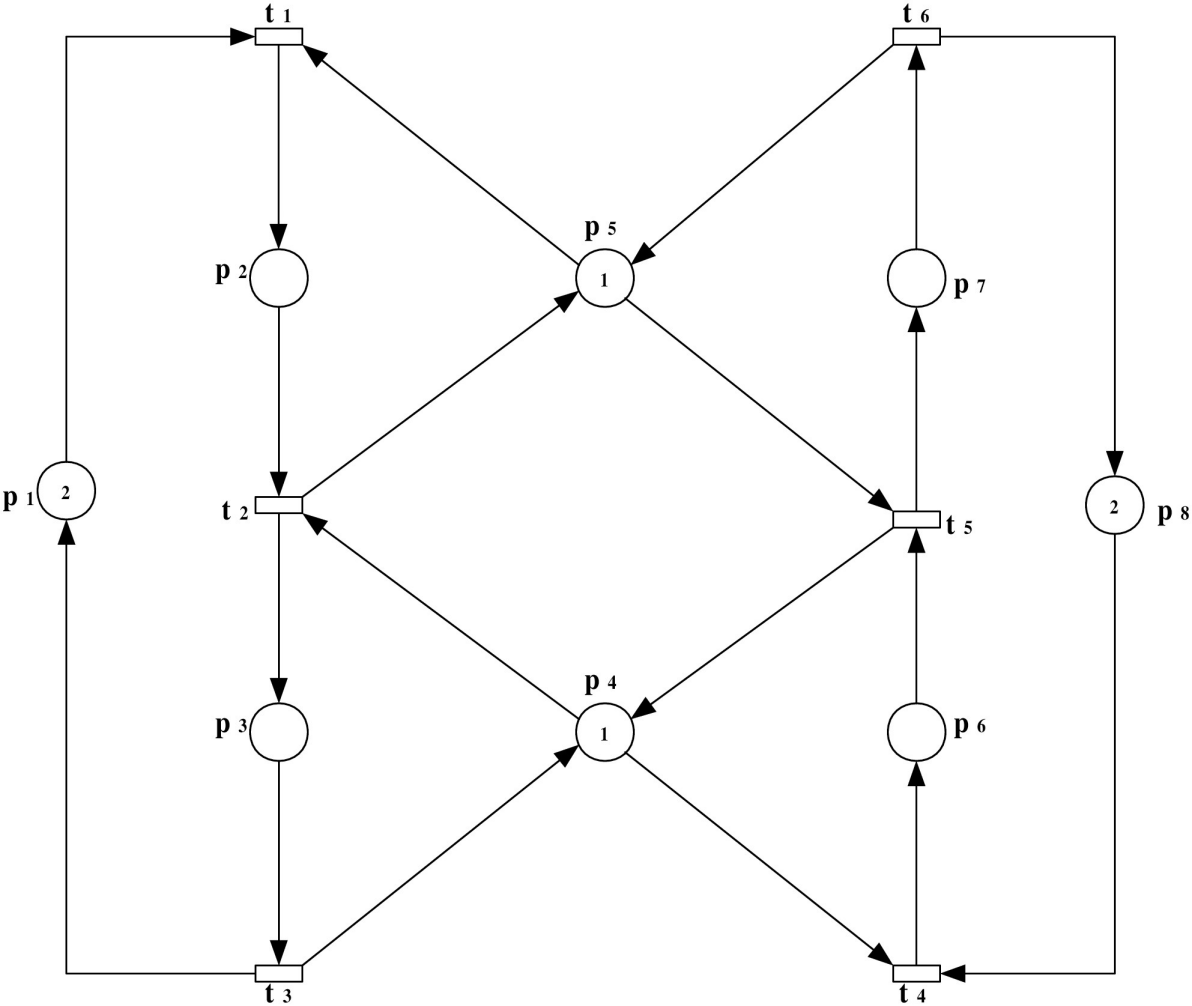
PN model $\left(N,M_{0}\right)$.

**Example 2**: For a bounded PN model, denoted as $\left(N,M_{0}\right)$ with N=(P,T,F,W), as illustrated in Fig 6, we investigate the scenario of an insertion attack at transition *t*_1_. We will study this example step by step as follows:

***Step 1***: *Scenario of an insertion attack and the observation system augmentation*

**Fig 7:**
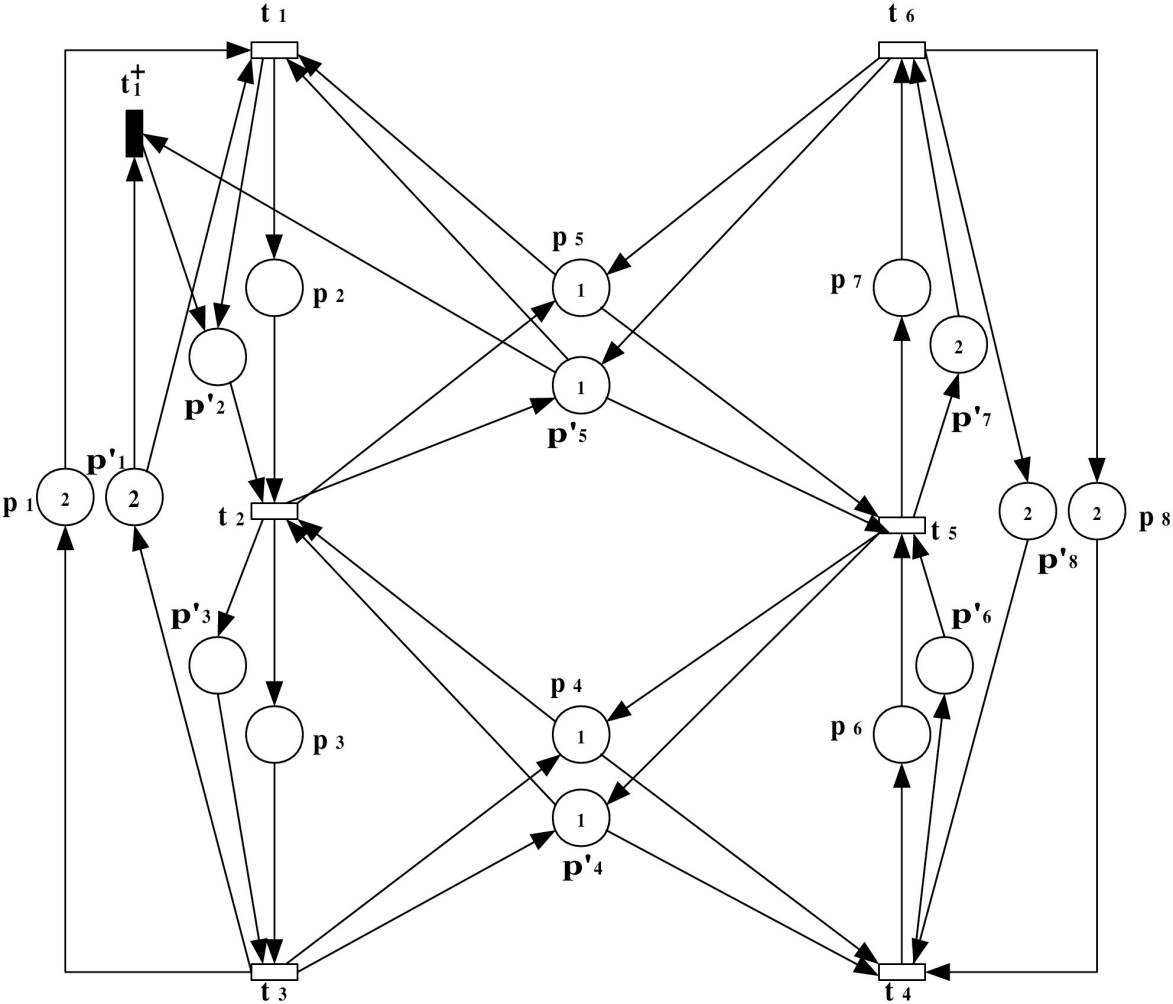
The observation system $\left(N^{\prime \prime },M_{0}^{\prime }\right)$ with an insertion attack transition $t_{1}^{+}$ that occurred at transition *t*_1_.

In this step, a scenario of an insertion attack at a specific transition within the PN model is introduced. The augmented observation system is developed to analyze the impact of the attack on the system’s behavior, highlighting the altered flow and resource allocation. The augmented observation system for the model under attack is represented as $\left(N^{\prime \prime },M_{0}^{\prime }\right)$. This system is characterized by $N^{\prime \prime }=\left(\bar{P},T_{\text{attack}}=T\cup \left\{t_{1}^{+}\right\},F_{\text{attack}}=\bar{F}\cup {\bar{F}}_{\text{in}},\bar{W}\right)$, as depicted in Fig 7.

***Step 2***: *ERG generation*

**Fig 8:**
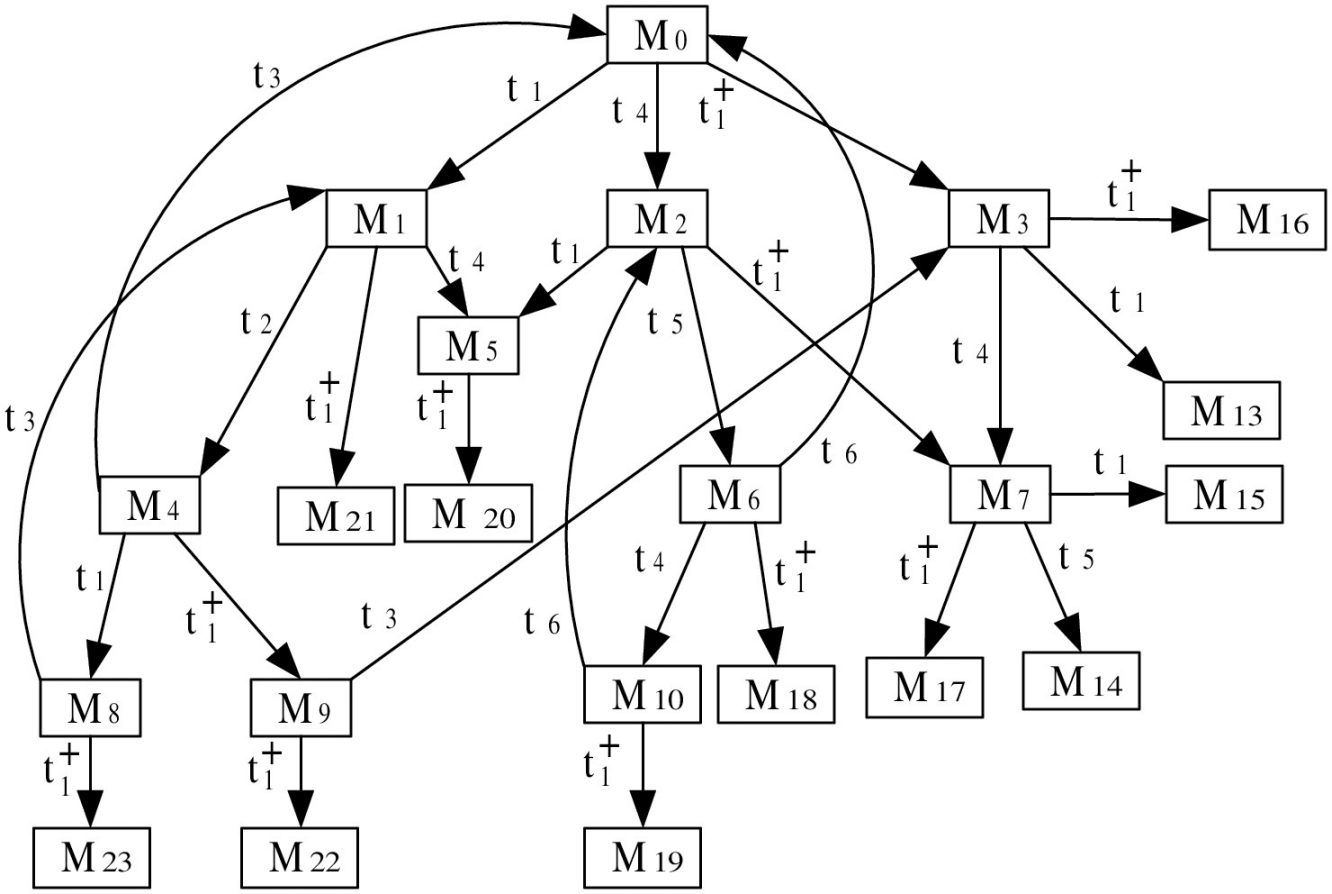
The ERG of $\left(N^{\prime \prime },M_{0}^{\prime }\right)$.

This step explains the generation of the ERG derived from the augmented PN model under attack. The ERG provides a comprehensive view of the system’s states and transitions, illustrating how the attack influences normal operation. The ERG derived from the observation system $\left(N^{\prime \prime },M_{0}^{\prime }\right)$ is presented in Fig 8, offering a thorough analysis of the system’s behavior under the specified attack scenario.

The marking of the state *M* can be described according to the general expression for multiset as explained in Example 1 (details shown in S1 File in S1 Data). The markings in Fig 8 are presented as follows:

**Markings**

$$\begin{array}{cc} M_{0} & 2p_{1}+p_{4}+p_{5}+2p_{8}+2p_{1}^{\prime }+p_{4}^{\prime }+{p^{\prime }}_{5}+2p_{8}^{\prime }\\ M_{1} & p_{1}+p_{2}+p_{4}+2p_{8}+p_{1}^{\prime }+p_{2}^{\prime }+p_{4}^{\prime }+2p_{8}^{\prime }\\ M_{2} & 2p_{1}+p_{5}+p_{6}+p_{8}+2{p^{\prime }}_{1}+{p^{\prime }}_{5}+{p^{\prime }}_{6}+{p^{\prime }}_{8}\\ M_{3} & 2p_{1}+p_{4}+p_{5}+2p_{8}+p_{1}^{\prime }+p_{2}^{\prime }+p_{4}^{\prime }+2p_{8}^{\prime }\\ M_{4} & p_{1}+p_{3}+p_{5}+2p_{8}+{p^{\prime }}_{1}+{p^{\prime }}_{3}+p_{5}^{\prime }+2p_{8}\\ M_{5} & p_{1}+p_{2}+p_{6}+p_{8}+p_{11}+{p^{\prime }}_{2}+{p^{\prime }}_{6}+{p^{\prime }}_{8}\\ M_{6} & 2p_{1}+p_{4}+p_{7}+p_{8}+2{p^{\prime }}_{1}+{p^{\prime }}_{4}+{p^{\prime }}_{7}+{p^{\prime }}_{8}\\ M_{7} & 2p_{1}+p_{5}+p_{6}+p_{8}+{p^{\prime }}_{1}+{p^{\prime }}_{2}+{p^{\prime }}_{6}+{p^{\prime }}_{8}\\ M_{8} & p2+p_{3}+2p_{8}+{p^{\prime }}_{2}+{p^{\prime }}_{3}+2{p^{\prime }}_{8}\\ M_{9} & p_{1}+p_{3}+p_{5}+2p_{8}+{p^{\prime }}_{2}+{p^{\prime }}_{3}+2p^{\prime }8\\ M_{10} & 2p_{1}+p_{6}+p_{7}+2p_{1}^{\prime }+{p^{\prime }}_{6}+p_{7}^{\prime }\\ M_{13} & p_{1}+p_{2}+p_{4}+2p_{8}+2{p^{\prime }}_{2}+p_{4}^{\prime }-p_{5}^{\prime }+2p_{8}^{\prime }\\ M_{14} & 2p_{1}+p_{4}+p_{7}+p_{8}+{p^{\prime }}_{1}+{p^{\prime }}_{2}+{p^{\prime }}_{4}-{p^{\prime }}_{5}+{p^{\prime }}_{7}+{p^{\prime }}_{8}\\ M_{15} & p_{1}+p_{2}+p_{6}+p_{8}+2{p^{\prime }}_{2}-{p^{\prime }}_{5}+{p^{\prime }}_{6}+{p^{\prime }}_{8}\\ M_{16} & 2p_{1}+p_{4}+p_{5}+2p_{8}+2p_{2}^{\prime }+p_{4}^{\prime }-{p^{\prime }}_{5}+2p_{8}^{\prime }\\ M_{17} & 2p_{1}+p_{5}+p_{6}+p_{8}+2{p^{\prime }}_{2}-{p^{\prime }}_{5}+{p^{\prime }}_{6}+{p^{\prime }}_{8}\\ M_{18} & 2p_{1}+p_{4}+p_{7}+p_{8}+{p^{\prime }}_{1}+{p^{\prime }}_{2}+{p^{\prime }}_{4}-{p^{\prime }}_{5}+{p^{\prime }}_{7}+{p^{\prime }}_{8}\\ M_{19} & 2p_{1}+p_{6}+p_{7}+p_{1}^{\prime }+{p^{\prime }}_{2}-{p^{\prime }}_{5}+{p^{\prime }}_{6}+p_{7}^{\prime }\\ M_{20} & p_{1}+p_{2}+p_{6}+p_{8}+p_{11}-{p^{\prime }}_{1}+2{p^{\prime }}_{2}-{p^{\prime }}_{5}+{p^{\prime }}_{6}+{p^{\prime }}_{8}\\ M_{21} & p_{1}+p_{2}+p_{4}+2p_{8}+2p_{2}^{\prime }+p_{4}^{\prime }-p_{5}^{\prime }+2p_{8}^{\prime }\\ M_{22} & p_{1}+p_{3}+p_{5}+2p_{8}-{p^{\prime }}_{1}+2{p^{\prime }}_{2}+{p^{\prime }}_{3}-{p^{\prime }}_{5}+2{p^{\prime }}_{8}\\ M_{23} & p2+p_{3}+2p_{8}-{p^{\prime }}_{1}+2{p^{\prime }}_{2}+{p^{\prime }}_{3}-{p^{\prime }}_{5}+2{p^{\prime }}_{8} \end{array}$$

***Step 3***: *ILPP formulation*

The ILPP is formulated in this step to address the occurrence of negative markings resulting from the attack. In this scenario, the occurrence of negative markings *M*_23_, *M*_22_, *M*_21_, *M*_20_, *M*_19_, *M*_18_, *M*_17_, *M*_16_, *M*_15_, *M*_14_, and *M*_13_ signifies the presence of an attack. The association between an identification place *p*_*s*_ and transitions is conceptualized through the equation *x*_*i*:_[*N*_*s*_](*p*_*s*_) = [*x*_1_ at *t*_1_ and $t_{1}^{+},x_{2}$ at *t*_2_, *x*_3_ at *t*_3_, *x*_4_ at *t*_4_, *x*_5_ at *t*_5_, *x*_6_ at *t*_6_]. For any nonnegative marking *M*, it holds *M*(*p*_*s*_)≥*b*. Hence, we have the following reachability conditions:

$$\begin{array}{c} M_{0}\left(p_{s}\right)\geq b,\\ M_{1}\left(p_{s}\right)=M_{3}\left(p_{s}\right)=M_{0}\left(p_{s}\right)+x_{1}\geq b,\\ M_{2}\left(p_{s}\right)=M_{0}\left(p_{s}\right)+x_{4}\geq b,\\ M_{4}\left(p_{s}\right)=M_{0}\left(p_{s}\right)+x_{1}+x_{2}\geq b,\\ M_{5}\left(p_{s}\right)=M_{7}\left(p_{s}\right)=M_{0}\left(p_{s}\right)+x_{1}+x_{4}\geq b,\\ M_{6}\left(p_{s}\right)=M_{0}\left(p_{s}\right)+x_{4}+x_{5}\geq b,\\ M_{8}\left(p_{s}\right)=M_{9}\left(p_{s}\right)=M_{0}\left(p_{s}\right)+2x_{1}+x_{2}\geq b,\\ M_{10}\left(p_{s}\right)=M_{0}\left(p_{s}\right)+2x_{4}+x_{5}\geq b, \end{array}$$

In this example, the cycle in the ERG involves the transitions sequence *t*_1_ → *t*_2_ → *t*_3_ initiating and concluding at the same node (beginning at *M*_0_) and *t*_4_ → *t*_5_ → *t*_6_ initiating and concluding at the same node (beginning at the *M*_0_). The cycle equations are:

$$\begin{array}{c} x_{3}+x_{2}+x_{1}=0,\\ x_{6}+x_{5}+x_{4}=0, \end{array}$$

For the negative markings *M*_23_, *M*_22_, *M*_21_, *M*_20_, *M*_19_, *M*_18_, *M*_17_, *M*_16_, *M*_15_, *M*_14_, and *M*_13_, it comes:

$$\begin{array}{c} M_{13}\left(p_{s}\right)=M_{16}\left(p_{s}\right)=M_{21}\left(p_{s}\right)=M_{0}\left(p_{s}\right)+2x_{1}\leq b-1,\\ M_{14}\left(p_{s}\right)=M_{18}\left(p_{s}\right)=M_{0}\left(p_{s}\right)+x_{1}+x_{4}+x_{5}\leq b-1,\\ M_{15}\left(p_{s}\right)=M_{17}\left(p_{s}\right)=M_{20}\left(p_{s}\right)=M_{0}\left(p_{s}\right)+2x_{1}+x_{4}\leq b-1,\\ M_{19}\left(p_{s}\right)=M_{0}\left(p_{s}\right)+x_{1}+2x_{4}+x_{5}\leq b-1,\\ M_{22}\left(p_{s}\right)=M_{23}\left(p_{s}\right)=M_{0}\left(p_{s}\right)+3x_{1}+x_{2}\leq b-1. \end{array}$$

An objective function is proposed to minimize *M*_0_(*p*_*s*_) in this example. By solving the obtained ILPP (details shown in S1 File in S1 Data), an optimal solution with *M*_0_(*p*_*s*_) = 1, *b* = 0, and $\left[N_{p_{s}}\right]=\left[-1,1,0,0,-1,1\right]$ is obtained.

***Step 4***: *Verification paths*

Verification paths are examined to ensure that under attack conditions, the system’s transitions lead to safe states. This step focuses on validating the system’s robustness by analyzing the sequences of markings and transitions under normal and compromised conditions. The markings and transitions sequences in Table 2 show that for any marking *M* with *M*(*p*_*s*_) ≤ *b* − 1, there does not exist any path without attack from *M*_0_ to *M* such as *M*_23_, *M*_22_, *M*_21_, *M*_20_, *M*_19_, *M*_18_, *M*_17_, *M*_16_, *M*_15_, *M*_14_, and *M*_13_.

**Table 2:** Verification paths.

Markings	Paths $\left(t_{1}t_{1}^{+}t_{2}t_{3}t_{4}t_{5}t_{6}\right)$	Markings	Paths $\left(t_{1}t_{1}^{+}t_{2}t_{3}t_{4}t_{5}t_{6}\right)$
*M*_0_	0000000	*M*_13_	1111100
*M*_1_	1000000	*M*_14_	1111101
*M*_2_	0100000	*M*_15_	1110000
*M*_3_	1010000	*M*_16_	1111000
*M*_4_	0200000	*M*_17_	1100000
*M*_5_	1011000	*M*_18_	1111100
*M*_6_	1011100	*M*_19_	1111101
*M*_7_	1011101	*M*_20_	1110000
*M*_8_	1100000	*M*_21_	1111000
*M*_9_	1100000	*M*_22_	1110000
*M*_10_	1100000	*M*_23_	1111000

**Example 3**: This example describes an automated manufacturing system (AMS) involving a single robot (*R*_1_) and a machine (*F*_1_). The system’s behavior is modeled by a PN to illustrate the movement of parts, the availability of resources, and the effects of an insertion attack on the system.

***Step 1***: *AMS description and its PN model representation*

**Fig 9:**
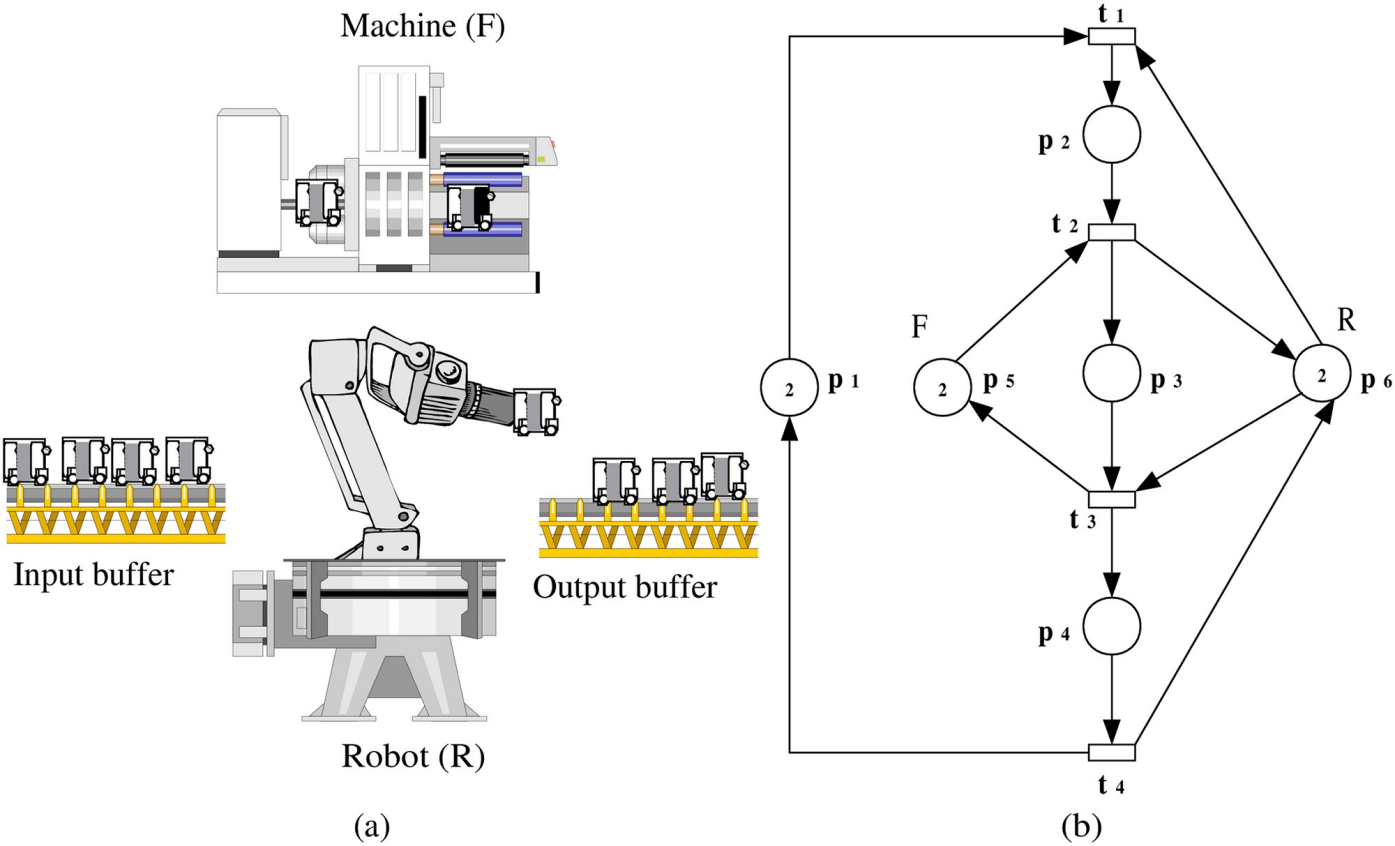
(a) Manufacturing system and (b) its PN model $\left(N,M_{0}\right)$.

This step involves a detailed description of the AMS and its corresponding PN model. The focus is on how the system components, such as the robot and machine, are represented within the PN framework to simulate the operational workflow. Here, we consider an AMS illustrated in Fig 9(a). The system includes a single robot (*R*_1_) and a machine (*F*_1_). Machine (*F*_1_) is responsible for processing one part at a time, and robot *R*_1_ is capable of holding and transporting one part at a time between various stages of the process. The system is equipped with input and output buffers that facilitate the loading and unloading of parts. In this setup, only one type of part is considered for processing throughout the system. In this model, places are used to represent the status of resources (robot and machine), the ongoing operations (processing or transportation), and the activities occurring within the system. The transitions correspond to control changes, depicting how the system evolves from one state to another as parts move through the manufacturing process. Directed arcs in the PN indicate the flow of materials, resources, and information, guiding the transitions between different states. Tokens within the PN represent these materials, resources, and information, moving between places as transitions fire, thus simulating the dynamic behavior of the manufacturing system. The PN model of this system, as shown in Fig 9(b), consists of six places and four transitions. These places represent different states in the system, including the availability of resources, the progress of operations, and the activities performed by the system. The transitions in the model signify changes in the state of the system, such as the movement of a part from one stage of processing to another. The initial marking of the PN is given as *M*_*o*_ = (2, 0, 0, 0, 2, 2)^*T*^. This marking indicates the initial distribution of parts and the availability of resources within the system. For instance, two tokens in place *p*_1_ signify that there are two raw parts available for processing. The tokens in *p*_5_ and *p*_6_ represent the availability of the robot and machine, respectively.

***Step 2***: *Scenario of an insertion attack and the observation system augmentation*

**Fig 10:**
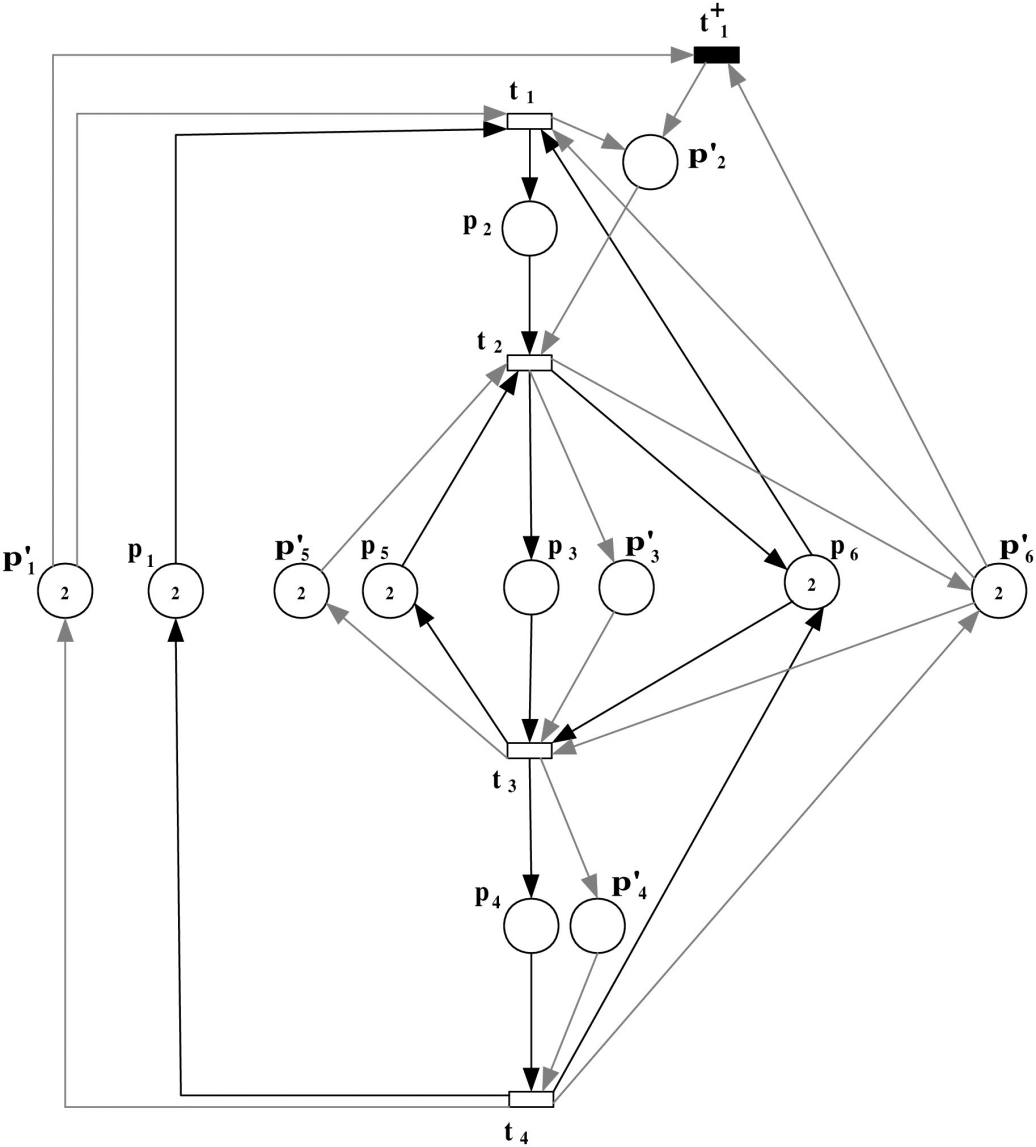
The observation system $\left(N^{\prime \prime },M_{0}^{\prime }\right)$ with an insertion attack transition $t_{1}^{+}$ that occurred at transition *t*_1_.

In this step, a scenario of an insertion attack at a specific transition within the PN model is introduced. The augmented observation system is developed to analyze the impact of the attack on the system’s behavior, highlighting the altered flow and resource allocation. For a bounded PN model $\left(N,M_{0}\right)$ with N=(P,T,F,W), illustrated in Fig 9(b), we investigate the scenario of an insertion attack at transition *t*_1_. The augmented observation system for the model under attack is represented as $\left(N^{\prime \prime },M_{0}^{\prime }\right)$. This system is characterized by $N^{\prime \prime }=\left(\bar{P},T_{\text{attack}}=T\cup \left\{t_{1}^{+}\right\},F_{\text{attack}}=\bar{F}\cup {\bar{F}}_{\text{in}},\bar{W}\right)$, as depicted in Fig 10.

***Step 3***: *ERG generation*

**Fig 11:**
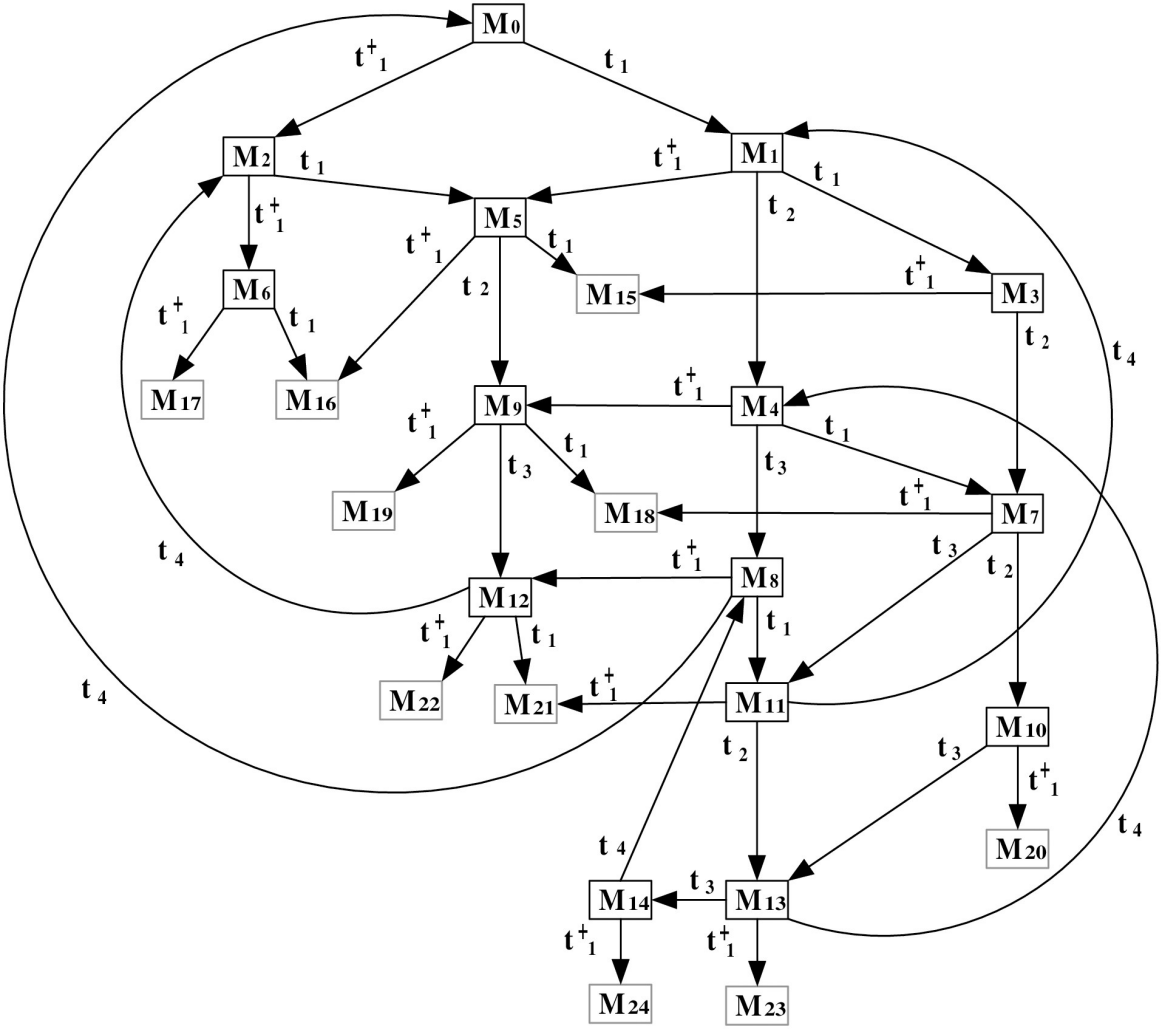
The ERG of $\left(N^{\prime \prime },M_{0}^{\prime }\right)$.

This step explains the generation of the ERG derived from the augmented PN model under attack. The ERG provides a comprehensive view of the system’s states and transitions, illustrating how the attack influences normal operation. The ERG derived from the observation system $\left(N^{\prime \prime },M_{0}^{\prime }\right)$ is presented in Fig 11, offering a thorough analysis of the system’s behavior under the specified attack scenario. The marking of the state *M* can be described according to the general expression for multiset as explained in Example 1 (details shown in S1 File in S1 Data). The markings in Fig 11 are presented as follows:

**Markings**

$$\begin{array}{cc} M_{0} & 2p_{1}+2p_{5}+2p_{6}+2{p^{\prime }}_{1}+2{p^{\prime }}_{5}+2{p^{\prime }}_{6}\\ M_{1} & p_{1}+p_{2}+2p_{5}+p_{6}+{p^{\prime }}_{1}+{p^{\prime }}_{2}+2{p^{\prime }}_{5}+{p^{\prime }}_{6}\\ M_{2} & 2p_{1}+2p_{5}+2p_{6}+{p^{\prime }}_{1}+{p^{\prime }}_{2}+2{p^{\prime }}_{5}+{p^{\prime }}_{6}\\ M_{3} & 2p_{2}+2p_{5}+2{p^{\prime }}_{2}+2{p^{\prime }}_{5}\\ M_{4} & p_{1}+p_{3}+p_{5}+2p_{6}+{p^{\prime }}_{1}+{p^{\prime }}_{3}+{p^{\prime }}_{5}+2{p^{\prime }}_{6}\\ M_{5} & p_{1}+p_{2}+2p_{5}+p_{6}+2{p^{\prime }}_{2}+2{p^{\prime }}_{5}\\ M_{6} & 2p_{1}+2p_{5}+2p_{6}+2{p^{\prime }}_{2}+2{p^{\prime }}_{5}\\ M_{7} & p_{2}+p_{3}+p_{5}+p_{6}+{p^{\prime }}_{2}+{p^{\prime }}_{3}+{p^{\prime }}_{5}+{p^{\prime }}_{6}\\ M_{8} & p_{1}+p_{4}+2p_{5}+p_{6}+{p^{\prime }}_{1}+{p^{\prime }}_{4}+2{p^{\prime }}_{5}+{p^{\prime }}_{6}\\ M_{9} & p_{1}+p_{3}+p_{5}+2p_{6}+{p^{\prime }}_{2}+{p^{\prime }}_{3}+{p^{\prime }}_{5}+{p^{\prime }}_{6}\\ M_{10} & 2p_{3}+2p_{6}+2{p^{\prime }}_{3}+2{p^{\prime }}_{6}\\ M_{11} & p_{2}+p_{4}+2p_{5}+{p^{\prime }}_{2}+{p^{\prime }}_{4}+2{p^{\prime }}_{5}\\ M_{12} & p_{1}+p_{4}+2p_{5}+p_{6}+{p^{\prime }}_{2}+{p^{\prime }}_{4}+2{p^{\prime }}_{5}\\ M_{13} & p_{3}+p_{4}+p_{5}+p_{6}+{p^{\prime }}_{3}+{p^{\prime }}_{4}+{p^{\prime }}_{5}+{p^{\prime }}_{6}\\ M_{14} & 2p_{4}+2p_{5}+2{p^{\prime }}_{4}+2{p^{\prime }}_{5}\\ M_{15} & 2p_{2}+2p_{5}-{p^{\prime }}_{1}+3{p^{\prime }}_{2}+2{p^{\prime }}_{5}-{p^{\prime }}_{6}\\ M_{16} & p_{1}+p_{2}+2p_{5}+p_{6}-{p^{\prime }}_{1}+3{p^{\prime }}_{2}+2{p^{\prime }}_{5}-{p^{\prime }}_{6}\\ M_{17} & 2p_{1}+2p_{5}+2p_{6}-{p^{\prime }}_{1}+3{p^{\prime }}_{2}+2{p^{\prime }}_{5}-{p^{\prime }}_{6}\\ M_{18} & p_{2}+p_{3}+p_{5}+p_{6}-{p^{\prime }}_{1}+2{p^{\prime }}_{2}+{p^{\prime }}_{3}+{p^{\prime }}_{5}\\ M_{19} & p_{3}+p_{5}+2p_{6}-{p^{\prime }}_{1}+2{p^{\prime }}_{2}+{p^{\prime }}_{3}+{p^{\prime }}_{5}\\ M_{20} & 2p_{3}+2p_{6}-{p^{\prime }}_{1}+{p^{\prime }}_{2}+2{p^{\prime }}_{3}+{p^{\prime }}_{6}\\ M_{21} & p_{2}+p_{4}+2p_{5}-{p^{\prime }}_{1}+2{p^{\prime }}_{2}+{p^{\prime }}_{4}+2{p^{\prime }}_{5}-{p^{\prime }}_{6}\\ M_{22} & p_{1}+p_{4}+2p_{5}+p_{6}-{p^{\prime }}_{1}+2{p^{\prime }}_{2}+{p^{\prime }}_{4}+2{p^{\prime }}_{5}-{p^{\prime }}_{6}\\ M_{23} & p_{3}+p_{4}+p_{5}+p_{6}-{p^{\prime }}_{1}+{p^{\prime }}_{2}+{p^{\prime }}_{3}+{p^{\prime }}_{4}+{p^{\prime }}_{5}\\ M_{24} & 2p_{4}+2p_{5}-{p^{\prime }}_{1}+{p^{\prime }}_{2}+2{p^{\prime }}_{4}+2{p^{\prime }}_{5}-{p^{\prime }}_{6} \end{array}$$

***Step 4***: *ILPP formulation*

The ILPP is formulated in this step to address the occurrence of negative markings resulting from the attack. In this scenario, the occurrence of negative markings *M*_15_, *M*_16_, *M*_17_, *M*_18_, *M*_19_, *M*_20_, *M*_21_, *M*_22_, *M*_23_, and *M*_24_ signifies the presence of an attack. The association between an identification place *p*_*s*_ and transitions is conceptualized through the equation *x*_*i*:_[*N*_*s*_](*p*_*s*_) = [*x*_1_ at *t*_1_ and $t_{1}^{+},x_{2}$ at *t*_2_, *x*_3_ at *t*_3_, *x*_4_ at *t*_4_]. For any nonnegative marking *M*, it holds *M*(*p*_*s*_)≥*b*. Hence, we have the following reachability conditions:

$$\begin{array}{c} M_{0}\left(p_{s}\right)\geq b,\\ M_{1}\left(p_{s}\right)=M_{2}\left(p_{s}\right)=M_{0}\left(p_{s}\right)+x_{1}\geq b,\\ M_{3}\left(p_{s}\right)=M_{5}\left(p_{s}\right)=M_{6}\left(p_{s}\right)=M_{0}\left(p_{s}\right)+2x_{1}\geq b,\\ M_{4}\left(p_{s}\right)=M_{0}\left(p_{s}\right)+x_{1}+x_{2}\geq b,\\ M_{7}\left(p_{s}\right)=M_{9}\left(p_{s}\right)=M_{0}\left(p_{s}\right)+2x_{1}+x_{2}\geq b,\\ M_{8}\left(p_{s}\right)=M_{0}\left(p_{s}\right)+x_{1}+x_{2}+x_{3}\geq b,\\ M_{10}\left(p_{s}\right)=M_{0}\left(p_{s}\right)+2x_{1}+2x_{2}\geq b,\\ M_{11}\left(p_{s}\right)=M_{12}\left(p_{s}\right)=M_{0}\left(p_{s}\right)+2x_{1}+x_{2}+x_{3}\geq b,\\ M_{13}\left(p_{s}\right)=M_{0}\left(p_{s}\right)+2x_{1}+2x_{2}+x_{3}\geq b,\\ M_{14}\left(p_{s}\right)=M_{0}\left(p_{s}\right)+2x_{1}+2x_{2}+2x_{3}\geq b, \end{array}$$

In this example, the cycle in the ERG involves the transitions sequence *t*_1_ → *t*_2_ → *t*_3_ → *t*_4_ initiating and concluding at the same node (beginning at *M*_0_, *M*_1_, *M*_2_, *M*_4_, and *M*_8_). The cycle equations are:

$$x_{1}+x_{2}+x_{3}+x_{4}=0,$$

For the negative markings *M*_15_, *M*_16_, *M*_17_, *M*_18_, *M*_19_, *M*_20_, *M*_21_, *M*_22_, *M*_23_, and *M*_24_, it comes:

$$\begin{array}{c} M_{15}\left(p_{s}\right)=M_{16}\left(p_{s}\right)=M_{17}\left(p_{s}\right)=M_{0}\left(p_{s}\right)+3x_{1}\leq b-1,\\ M_{18}\left(p_{s}\right)=M_{19}\left(p_{s}\right)=M_{0}\left(p_{s}\right)+3x_{1}+x_{2}\leq b-1,\\ M_{20}\left(p_{s}\right)=M_{0}\left(p_{s}\right)+3x_{1}+2x_{2}\leq b-1,\\ M_{21}\left(p_{s}\right)=M_{22}\left(p_{s}\right)=M_{0}\left(p_{s}\right)+3x_{1}+x_{2}+x_{3}\leq b-1,\\ M_{23}\left(p_{s}\right)=M_{0}\left(p_{s}\right)+3x_{1}+2x_{2}+x_{3}\leq b-1,\\ M_{24}\left(p_{s}\right)=M_{0}\left(p_{s}\right)+3x_{1}+2x_{2}+2x_{3}\leq b-1. \end{array}$$

An objective function is proposed to minimize *M*_0_(*p*_*s*_) in this example. By solving the obtained ILPP (details shown in S1 File in S1 Data), an optimal solution with *M*_0_(*p*_*s*_) = 2, *b* = 0, and $\left[N_{p_{s}}\right]=\left[-1,0,0,1\right]$ is obtained.

***Step 5***: *Verification paths*

Verification paths are examined to ensure that under attack conditions, the system’s transitions lead to safe states. This step focuses on validating the system’s robustness by analyzing the sequences of markings and transitions under normal and compromised conditions. The markings and transitions sequences in Table 3 show that for any marking *M* with *M*(*p*_*s*_) ≤ *b* − 1, there does not exist any path without attack from *M*_0_ to *M* such as *M*_15_, *M*_16_, *M*_17_, *M*_18_, *M*_19_, *M*_20_, *M*_21_, *M*_22_, *M*_23_, and *M*_24_.

**Table 3:** Verification paths.

Markings	Paths $\left(t_{1}t_{1}^{+}t_{2}t_{3}t_{4}\right)$	Markings	Paths $\left(t_{1}t_{1}^{+}t_{2}t_{3}t_{4}\right)$
*M*_0_	00000	*M*_13_	20210
*M*_1_	10000	*M*_14_	20220
*M*_2_	01000	*M*_15_	21000
*M*_3_	20000	*M*_16_	12000
*M*_4_	10100	*M*_17_	03000
*M*_5_	11000	*M*_18_	21100
*M*_6_	02000	*M*_19_	12100
*M*_7_	20100	*M*_20_	21200
*M*_8_	10110	*M*_21_	21110
*M*_9_	11100	*M*_22_	12110
*M*_10_	20200	*M*_23_	21210
*M*_11_	20110	*M*_24_	21220
*M*_12_	11110	−	−

***Step 6***: *Results discussion*

This final step discusses the overall findings from the PN model analysis, including the system’s behavior under normal operations and the impact of the insertion attack. The discussion emphasizes the importance of robust system design and highlights the potential vulnerabilities identified through the PN model. The PN model developed for the AMS effectively captures the intricate dynamics of resource allocation, process control, and material flow within the system. The analysis of the PN under normal and attack scenarios provides significant insights into the system’s behavior, robustness, and vulnerabilities.

*(a) System behavior under normal conditions*

Under the initial marking *M*_0_ = (2, 0, 0, 0, 2, 2)^*T*^, the PN model exhibits a well-coordinated sequence of operations, where the robot and machine resources are effectively utilized to process parts. The marking distribution indicates that there are two raw parts ready for processing, and both the robot and machine are initially available. The transitions represent the sequential control changes as the system processes parts from the loading buffer to the machine and subsequently to the unloading buffer. As the transitions fire, the system progresses through different states, effectively illustrating the interplay between the robot’s transportation tasks and the machine’s processing operations.

*(b) Impact of insertion attack on transition t_1_*

When an insertion attack is introduced at transition *t*_1_, the system’s behavior changes significantly. The augmented PN model, denoted as $\left(N^{\prime \prime },M_{0}^{\prime }\right)$, incorporates the effects of the attack by adding the transition $t_{1}^{+}$, which disrupts the normal flow of operations. The resulting ERG provides a visual representation of how the system’s states are altered due to the attack. The occurrence of negative markings, such as *M*_15_ through *M*_24_, is a direct consequence of the attack. These negative markings indicate an abnormal state where the system’s resource and material flow are compromised. Specifically, the negative values suggest that the attack has led to an overconsumption of resources or an improper transition sequence, causing the system to reach an unreachable or unsafe state under normal operation.

*(c) Analysis of marking sequences and path verification*

The marking sequences *M*_15_ through *M*_24_ illustrate the presence of a significant deviation from the expected behavior of the AMS. The paths associated with these markings, particularly under the attack sequence $t_{1}\to t_{1}^{+}\to t_{2}\to t_{3}\to t_{4}$, reveal that the system when under attack, fails to maintain the proper balance of resources and parts. This highlights the critical nature of the attack on transition *t*_1_ and its impact on the system’s overall functionality. The cycle equations derived from the ERG further reinforce the idea that the system, in its compromised state, is unable to return to a safe and stable condition without external intervention. The cycle equation *x*_1_ + *x*_2_ + *x*_3_ + *x*_4_ = 0 underlines the disruption in the normal flow of operations caused by the attack.

*(d) Optimal solutions*

An ILPP is formulated to minimize the marking *M*_0_(*p*_*s*_). The optimal solution obtained suggests that by carefully adjusting the system’s parameters, it is possible to mitigate the effects of the attack and restore the system to a stable state. The optimal solution *M*_0_(*p*_*s*_) = 2 with *b* = 0 and the transition sequence $\left[N_{p_{s}}\right]=\left[-1,0,0,1\right]$ offers a potential recovery path for the system. The verification paths detailed in Table 3 show that for any marking *M* where *M*(*p*_*s*_) ≤ *b* − 1, no path without an attack exists that can transition the system from *M*_0_ to the negative markings *M*_15_ through *M*_24_. This indicates that under normal conditions, the system is resilient and able to prevent unsafe states, but the attack scenario exposes vulnerabilities that require attention.

The PN model of the AMS demonstrates its utility in simulating and analyzing the behavior of complex manufacturing systems. The results of this case study emphasize the importance of robust design and the potential risks posed by malicious attacks. By using PNs to model and analyze such systems, manufacturers can not only optimize normal operations but also develop strategies to detect and mitigate the effects of attacks, thereby enhancing the overall security and reliability of the manufacturing process.

## 6 Comparative analysis

In this section, we present a comparative analysis of the proposed method for insertion attack identification in DESs using PNs with an observer, contrasting it with existing approaches in the literature. The focus of this comparison is to highlight the advantages of the proposed methodology and identify any potential limitations.

*(a) Comparison with existing methods*

In Section 1, we provide a comprehensive overview of relevant works in the field, highlighting key differences between our approach to those proposed in previous research on PNs, FSA, DESs, and attack identification methodologies. Here, several methods have been developed for detecting and mitigating attacks in DESs, particularly those based on FSA and PNs. Existing literature has explored various approaches to model and detect attacks, such as using nondeterministic finite state transducers (NFST) [42], bipartite transition structures [44], and supervisory control frameworks under stealthy attack scenarios [33]. Compared to traditional FSA-based methods, the proposed approach offers a more compact and efficient framework for modeling DESs using PNs. While FSAs are effective in explicitly enumerating all possible system states, they often suffer from state explosion issues when dealing with complex systems with a large number of states [8, 23]. In contrast, PNs provide a more scalable solution, particularly in handling concurrent processes and asynchronous events [53].

Furthermore, the proposed method introduces a novel observation structure within the PN framework, enabling systematic modeling and identification of insertion attacks. This approach enhances the detection accuracy by generating an ERG that incorporates the observation structure, thereby identifying special markings indicative of attacks, including those with negative values. Unlike traditional methods that primarily focus on state estimation or diagnosability analysis [16, 17], the proposed method directly addresses the challenges posed by insertion attacks, providing a more targeted solution.

This research enhances detection accuracy by formulating an ILPP to compute the observation place where the number of tokens directly indicates an attack. This approach contrasts with earlier methods, such as those in [34, 40], which rely on diagnostic structures or algorithmic checks for possible control goal violations. By focusing on the token count within the PN model, our method ensures precise and immediate identification of insertion attacks.

*(b) Advantages of the proposed method*

The main advantages of the proposed method over the existing techniques can be summarized as follows:

1. By incorporating a novel observation structure within the PN framework, the proposed method provides a systematic approach to model insertion attacks, offering a more comprehensive representation of attack scenarios compared to traditional methods based on FSAs or standard PN models.
2. The method leverages an ERG that integrates the observation structure, allowing for the identification of unique markings indicative of attacks. It improves detection accuracy, particularly for identifying insertion attacks in systems with partial observations.
3. Unlike traditional FSA-based approaches, which suffer generally from state explosion issues, the proposed PN-based method is more scalable and efficient, making it suitable for complex DESs with large state spaces and numerous concurrent events.
4. The method utilizes an ILPP to design an optimal observation place within the PN model. This approach ensures efficient resource utilization and computational efficiency, which is particularly beneficial in real-world scenarios with limited computational resources. The ILPP-based design enhances the precision of attack identification by effectively leveraging token counts in the observation place.
5. The focus on token count changes in the observation place simplifies the monitoring process, improving detection efficiency and accuracy. This new practical approach reduces the complexity associated with detecting insertion attacks, making it easier to implement in applications where quick and reliable detection is essential.
6. The proposed approach is verified through practical examples, demonstrating its effectiveness in real-world scenarios. This practical validation enhances its applicability and reliability in different DES settings.

*(c) Potential limitations*

Despite its advantages, the proposed method has some potential limitations. The generation of an ERG that incorporates the observation structure can become computationally intensive, especially for very large and complex DESs. Additionally, while the method is specifically designed to address insertion attacks, it may need further adaptation to handle other types of attacks, such as deletion or substitution attacks, which are not directly covered in this study. Although the proposed method offers a novel and effective approach for identifying insertion attacks in DESs using PNs with enhanced modeling capabilities, improved detection accuracy, scalability, and practical applicability, it also presents challenges related to the assumptions about initial observations, the complexity of ERG generation, and its focus on a single type of attack. Future work could address these limitations by exploring methods to manage uncertainties in observations, optimizing the ERG generation process, and extending the framework to encompass a broader range of attack types.

Table 4 provides a comparative overview to compare the proposed method with several existing techniques for attack identification in DESs using PNs and related frameworks. This comparison focuses on highlighting the advantages and potential limitations of our approach against existing methods in the literature.

**Table 4:** Comparison of the proposed method with existing techniques for attack identification in DESs.

Aspect	Proposed Method	Wakaiki et al. (2019) [27]	Zhang et al. (2022) [51]	Fritz & Zhang (2018) [33]	Tong et al. (2022) [41]	Chen et al. (2021, 2022) [48, 49]
**Methodology**	Utilizes PNs with an observer and an ERG to model and detect insertion attacks	Employs modified automata and supervisory control theory with adversarial models without state expansion	Constructs an attack structure through parallel composition of an attacker observer and supervisor under attack	Uses permutation matrices to model and detect replay and covert attacks in CPS	Proposes an all sensor attack automaton to model all possible sensor attacks and verify conditions for threats	Iterative approach to separate admissible markings into subsets for optimal supervisor design with nonlinear constraints
**Focus of attack**	Insertion attacks on sensor data	Insertion and deletion of symbols in sensor outputs	Sensor and actuator attacks aiming to corrupt supervisor’s observations and enable events to reach unsafe states	CPS attacks, specifically replay and covert attacks	Sensor attacks that modify sensor readings to reach forbidden states	Attacks indirectly considered by ensuring optimal control and constraint separation in PNs
**Model used**	PNs with novel observation structure and ILP	DES with modified automata output maps	DES using attack structure automaton	CPS modeled as DESs	All sensor attack automaton for supervised DES	PNs with nonlinear constraints and subsets of admissible markings
**Detection mechanism**	Observation places computed via ILP detect negative markings indicating attacks	Controllability and a novel observability condition that incorporates adversaries	Parallel composition of automata to model attacker actions and identify paths to unsafe states	Permutation matrices to alter transmitted signals for detection of replay and covert attacks	Verification of stealthy threatening sensor attackers through polynomial time analysis of the ASA structure	Transformation of nonlinear constraints into conjunctive/disjunctive constraints, ensuring all markings are admissible
**Advantages**	High detection accuracy, scalable, systematically models insertion attacks	No state space expansion, applicable to multiple adversaries, utilizes existing DES tools	Comprehensive modeling of both sensor and actuator attacks, allows the supervisor to prevent reaching unsafe states	Addresses CPS security with a novel approach for difficult-to-detect deception attacks, applicable to complex systems	Polynomial-time verification of all possible sensor attacks, comprehensive threat model	Effective handling of nonlinear constraints in PNs, maximally permissive supervisor design
**Limitations**	Focused only on insertion attacks, may not generalize to other attack types	Limited to insertion and deletion attacks, assumes specific adversarial behaviors	Relies on precise modeling of attacker actions; may not account for all real-world attack scenarios	Focused on specific types of cyber attacks (replay and covert), may not cover other forms of attacks	Assumes a specific form of sensor attack, focuses on sensor attackers only	Assumes constraints can be separated into linear subsets, complexity may increase with nonlinear constraints

## 7 Conclusion

This paper presents a methodology for identifying sensor attacks in DESs using PNs. Sensor attacks often mislead observers, leading to incorrect decision-making. The focus is on one primary type of sensor attack namely insertion attacks. We develop a PN model incorporating these attack types and construct an observation structure to model these attacks within the system. The approach involves generating the ERG of the modified PN to identify a unique class of markings, which can assume negative values. An observation place within the PN is calculated by formulating and solving an ILP problem, capturing the dynamics of the system under insertion attack scenarios. The presence and intensity of an attack are deduced from the number of tokens in this observation place. Practical examples validate the proposed method, demonstrating its efficacy and applicability. In our future work, we will primarily focus on extending the methodology to handle complex, concurrent cyber-physical attack scenarios and integrating machine learning for enhanced predictive capabilities in dynamic systems.

## Supporting information

S1 Data (ZIP)
