# Supplementary material for: Insertion attack identification in discrete event systems using petri nets with an observer
Source: PLoS One. 2024 Dec 9;19(12):e0314104. doi: 10.1371/journal.pone.0314104 (PMC11627397; doi:10.1371/journal.pone.0314104)

Generation of the ERG for a system with its observation under an insertion attack and solving its ILPPs.

To generate the ERG, we will study the all markings for the observation system $(\mathcal{N}'', M_0')$ with an insertion attack transition t_1^+that occurred at transition t_1 and determines a set of markings that is divided into a set of negative markings $\mathcal{M}^-$ and a set of non-negative markings $\mathcal{M}^+$. Then we formulate and solving its ILPPs. We study the three examples mentioned in the paper as follow:

Example 1: The observation system $(\mathcal{N}'', M_0')$ as shown in Figure below, we will study the states of the system step by step according to the enabled transition as follow:

The system starts with the initial state $M_o = p_1 + p_3 + p'_1 + p'_3$

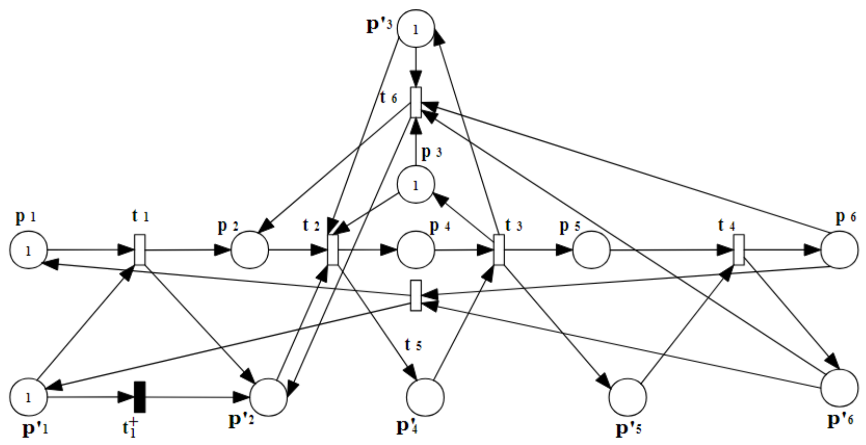

t_1 at M_0 is fired and the resulting marking is $M_1 = p_2 + p_3 + p'_2 + p'_3$

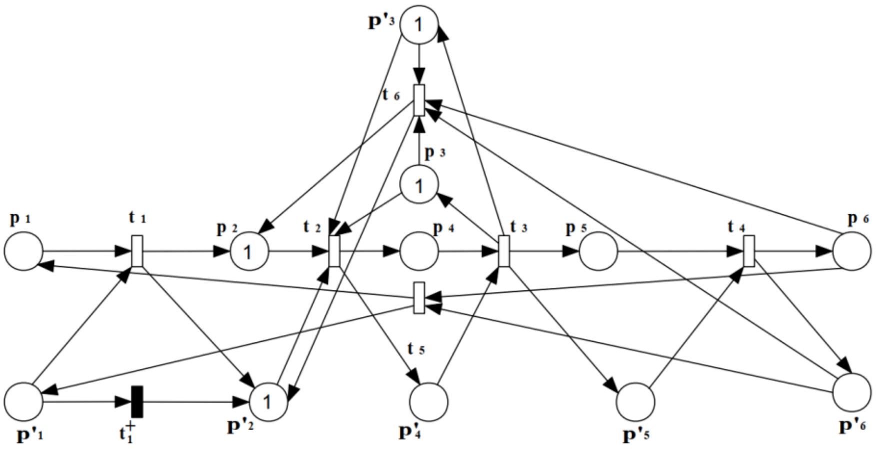

t_1^+at M_0 is fired and the resulting marking is $M_2 = p_1 + p_3 + p'_2 + p'_3$

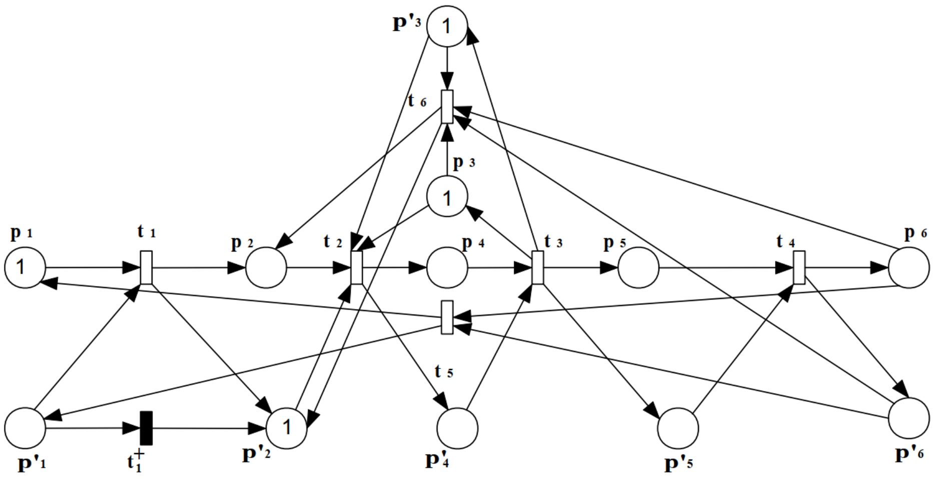

t_1^+ at M_2 is fired and the resulting marking is $M_3 = p_4 + p'_4$

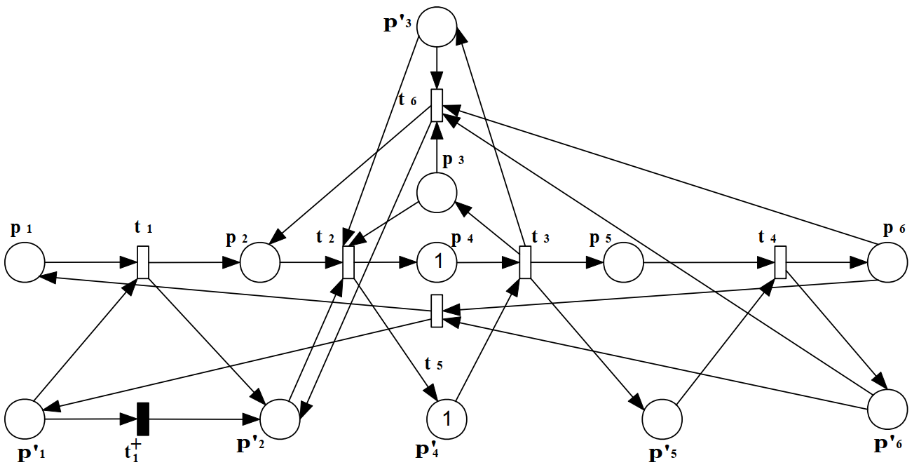

t_1^+ at M_0 is fired and the resulting marking is $M_4 = p_1 + p_3 - p'_1 + 2p'_2 + p'_3$

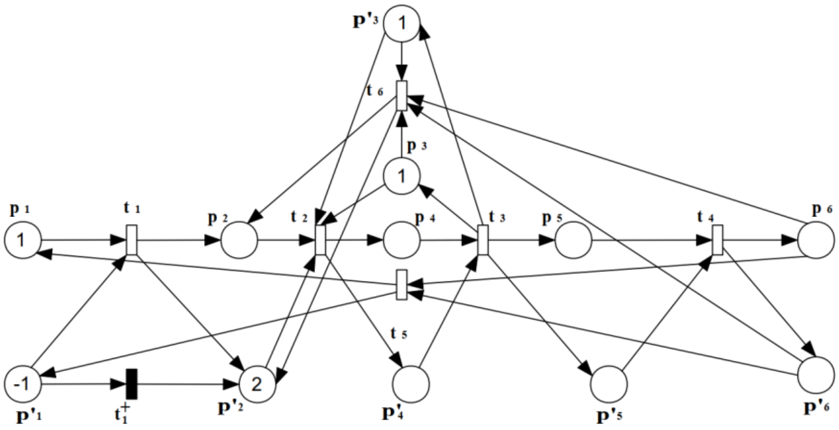

t_3 at M_3 is fired and the resulting marking is $M_5 = p_3 + p_5 + p'_3 + p'_5$

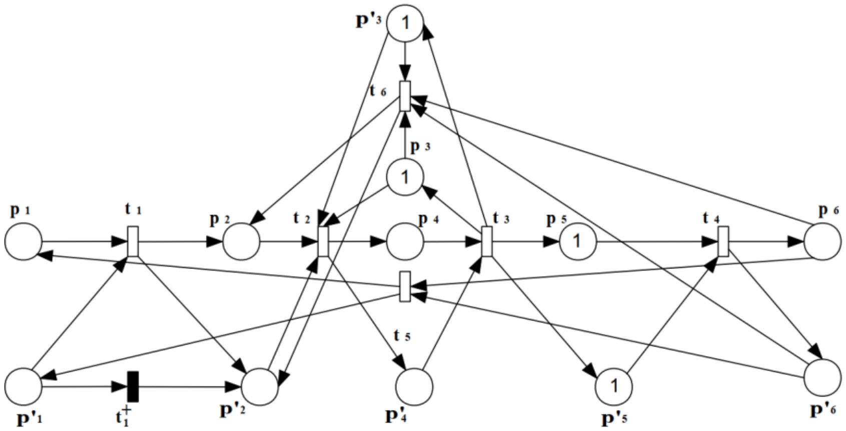

t_4 at M_5 is fired and the resulting marking is $M_6 = p_3 + p_6 + p'_3 + p'_6$

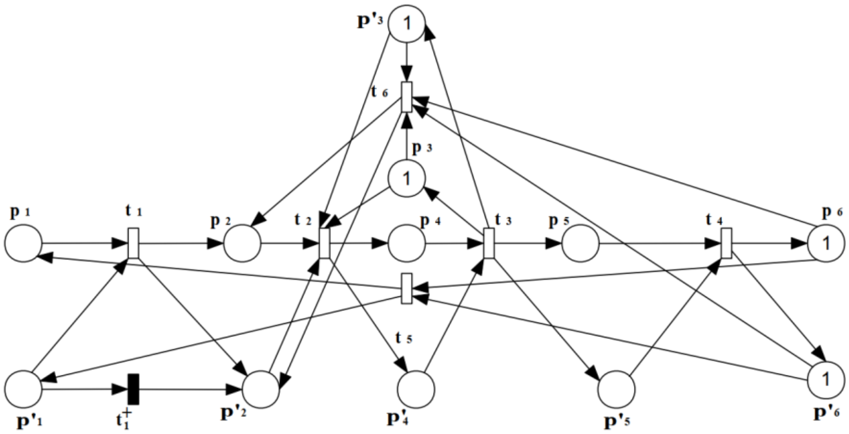

t_6 at M_6 is fired and the resulting marking is $M_7 = p_2 + p'_2$

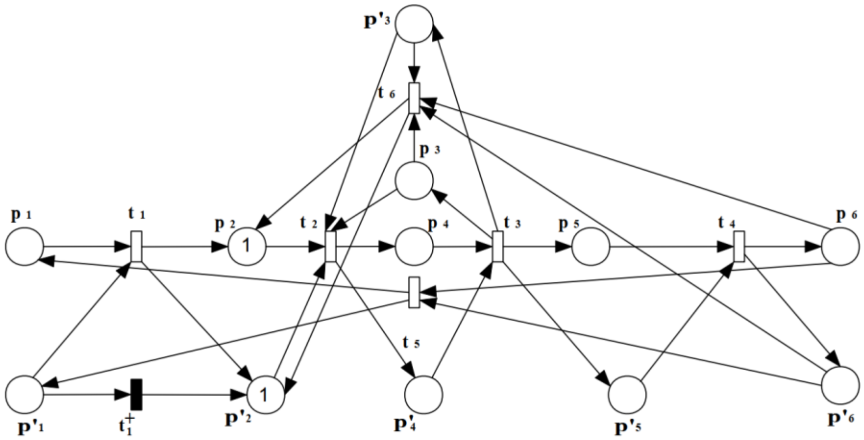

t_1 at M_2 is fired and the resulting marking is $M_9 = p_2 + p_3 - p'_1 + 2p'_2 + p'_3$

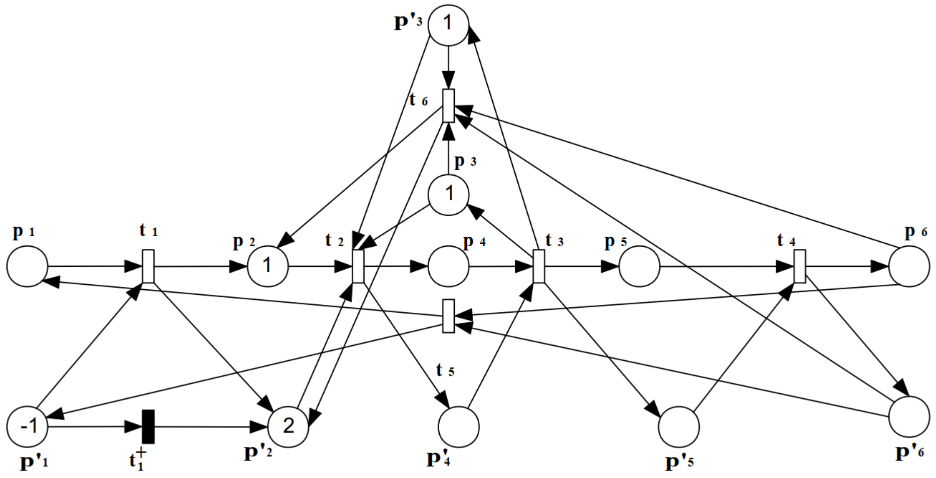

t_1^+ at M_1 is fired and the resulting marking is $M_{10} = p_2 + p_3 - p'_1 + 2p'_2 + p'_3$

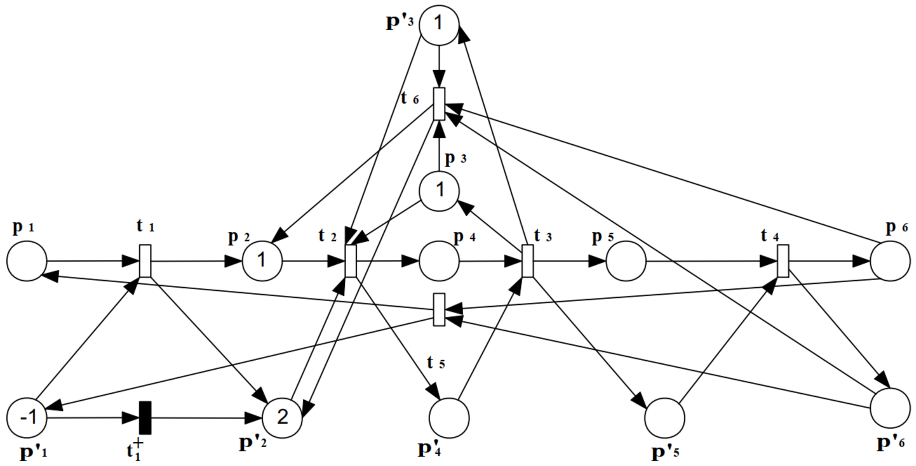

t_1^+ at M_3 is fired and the resulting marking is $M_{11} = p_4 - p'_1 + p'_2 + p'_4$

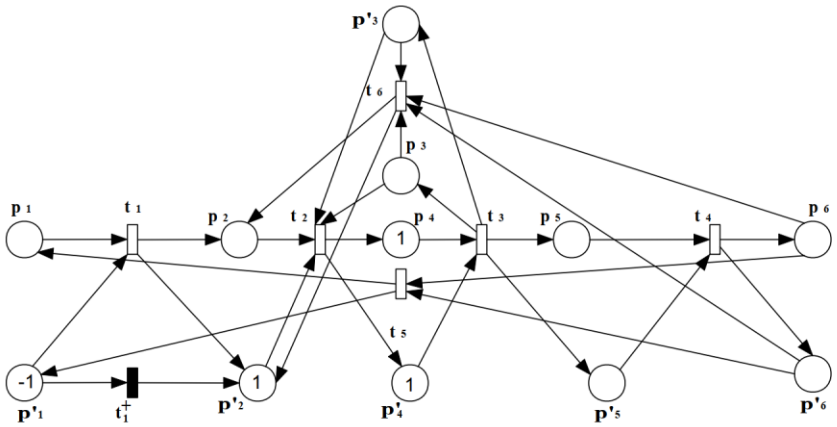

t_1^+ at M_5 is fired and the resulting marking is $M_{12} = p_3 + p_5 - p'_1 + p'_2 + p'_3 + p'_5$

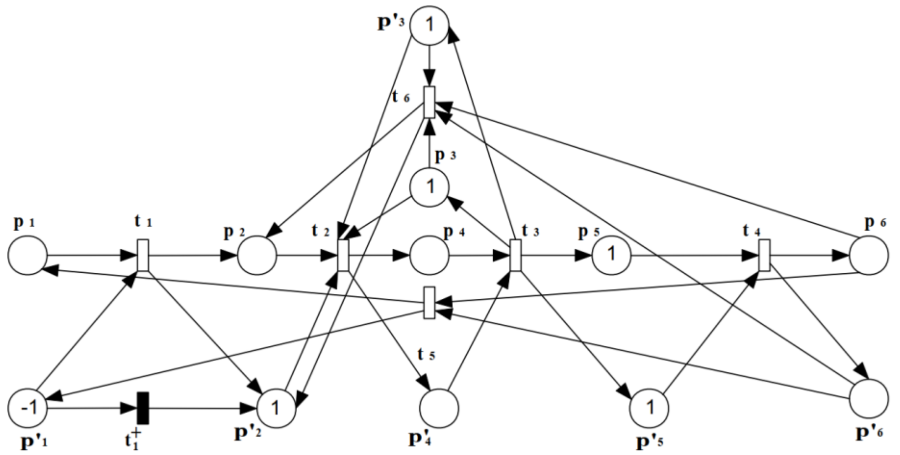

t_1^+ at M_6 is fired and the resulting marking is $M_{13} = p_3 + p_6 - p'_1 + p'_2 + p'_3 + p'_6$

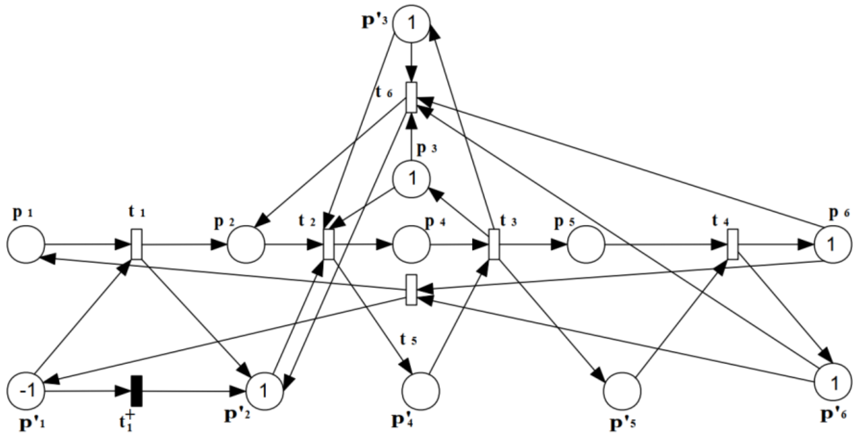

t_1^+ at M_7 is fired and the resulting marking is $M_{14} = p_2 - p'_1 + 2p'_2$

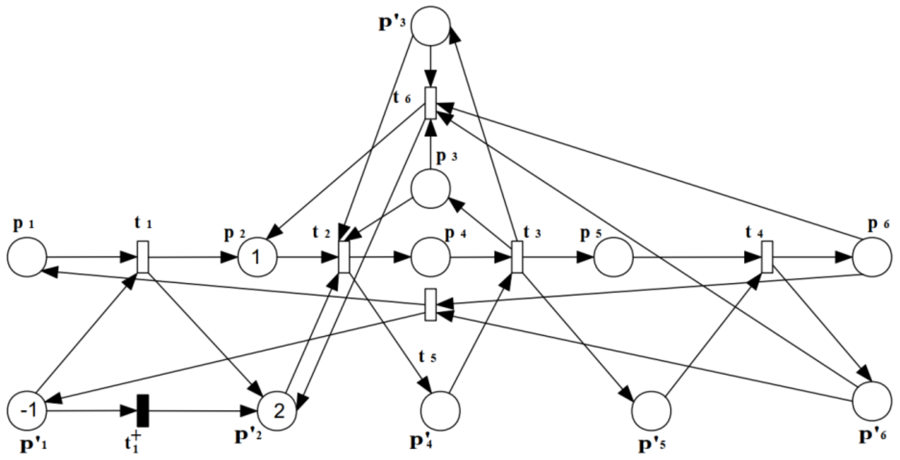

All these states in the system are included in the ERG as shown in below:

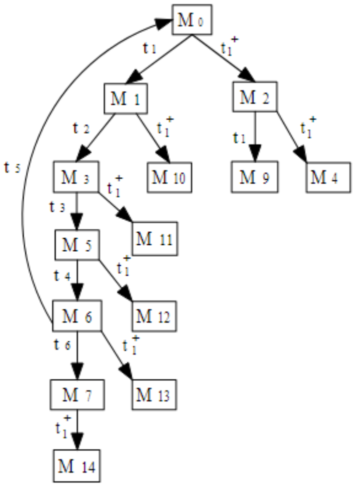

Formulate the ILPPs as shown in below:

```
Min=M;
M>=b;
M+y1-z1>=b;
M+y1-z1+y2-z2>=b;
M+y1-z1+y2-z2+y3-z3>=b;
M+y1-z1+y2-z2+y3-z3+y4-z4>=b;
M+y1-z1+y2-z2+y3-z3+y4-z4+y6-z6>=b;
y1-z1+y2-z2+y3-z3+y4-z4+y5-z5=0;
M+2*y1-2*z1<=b-1;
M+2*y1-2*z1+y2-z2<=b-1;
M+2*y1-2*z1+y2-z2+y3-z3<=b-1;
M+2*y1-2*z1+y2-z2+y3-z3+y4-z4<=b-1;
M+2*y1-2*z1+y2-z2+y3-z3+y4-z4+y6-z6<=b-1;
@gin(y1);
@gin(z1);
@gin(y2);
@gin(z2);
@gin(y3);
@gin(z3);
@gin(y4);
@gin(z4);
@gin(y5);
@gin(z5);
@gin(y6);
@gin(z6);
@gin(M);
@gin(b);
End
```

The ILPP is solved in the software Lingo as shown in the following figure.

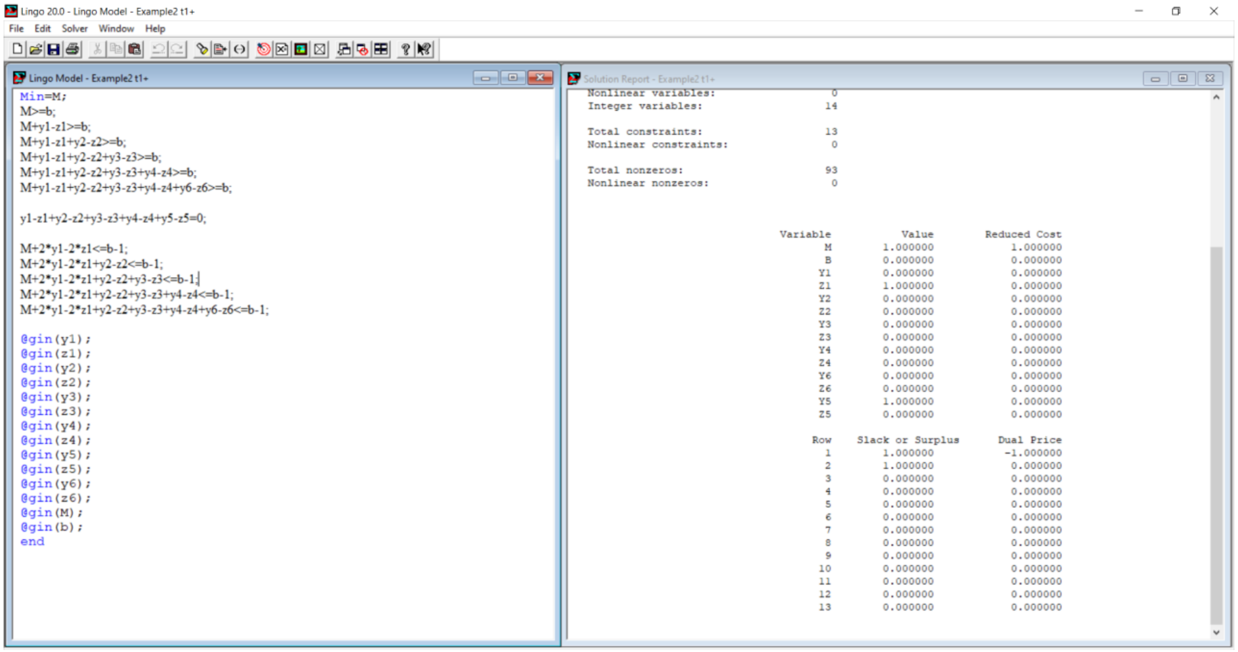

Example 2: The observation system $(\mathcal{N}'', M_0')$ as shown the figure below, we will study the states of the system step by step as follows:

The system starts with the initial marking $M_0 = 2p_1 + p_4 + p_5 + 2p_8 + 2p_1' + p_4' + p'\,_5 + 2p_8'$

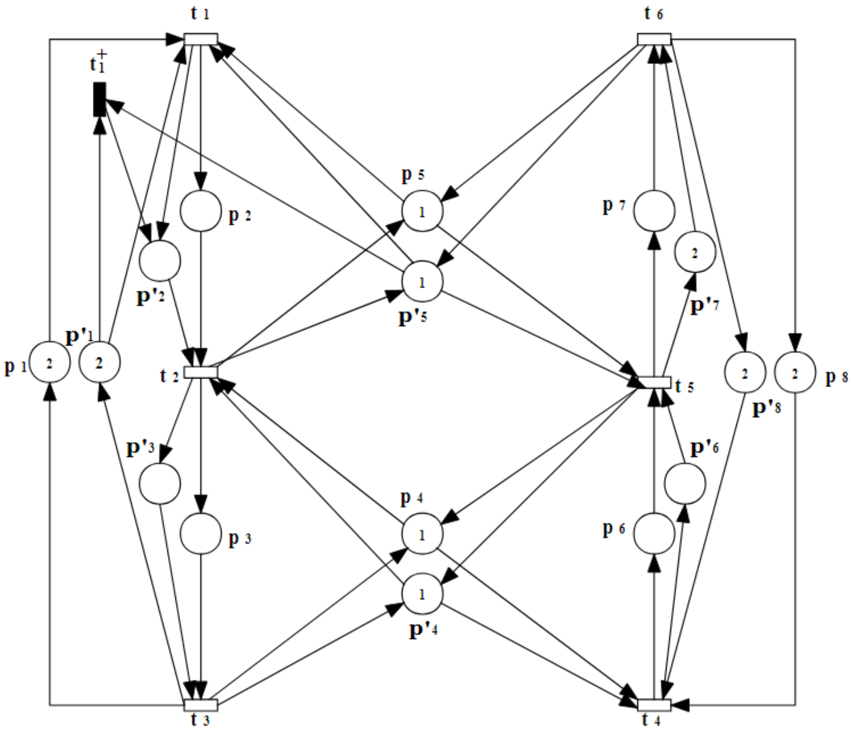

t_1 at M_0 is fired and the result is $M_1 = p_1 + p_2 + p_4 + 2p_8 + p_1' + p_2' + p_4' + 2p_8'$

t_4 at M_0 is fired and the resulting marking is $M_2 = 2p_1 + p_5 + p_6 + p_8 + 2p'_1 + p'_5 + p'_6 + p'_8$

t_1^+ at M_0 is fired and the resulting marking is $M_3 = 2p_1 + p_4 + p_5 + 2p_8 + p_1' + p_2' + p_4' + 2p_8'$

t_2 at M_1 is fired and the resulting marking is $M_4 = p_1 + p_3 + p_5 + 2p_8 + p'_1 + p'_3 + p_5' + 2p_8$

t_4 at M_1 or t_1 at M_2 is fired and the resulting marking is $M_5 = p_1 + p_2 + p_6 + p_8 + p_{11} + p'_2 + p'_6 + p'_8$

t_5 at M_2 is fired and the resulting marking is $M_6 = 2p_1 + p_4 + p_7 + p_8 + 2p'_1 + p'_4 + p'_7 + p'_8$

t_1^+ at M_2 or t_4 at $M_3$is fired and the resulting marking is $M_7 = 2p_1 + p_5 + p_6 + p_8 + p'_1 + p'_2 + p'_6 + p'_8$

t_1 at M_4 is fired and the resulting marking is $M_8 = p2 + p_3 + 2p_8 + p'_2 + p'_3 + 2p'_8$

t_1^+ at M_4 is fired and the resulting marking is $M_9 = p_1 + p_3 + p_5 + 2p_8 + p'_2 + p'_3 + 2p'_8$

t_4 at M_6 is fired and the resulting marking is $M_{10} = 2p_1 + p_6 + p_7 + 2p_1' + p'_6 + p_7'$

t_1 at M_3 is fired and the resulting marking is $M_{13} = p_1 + p_2 + p_4 + 2p_8 + 2p'_2 + p_4' - p_5' + 2p_8'$

t_5 at M_7 is fired and the resulting marking is $M_{14} = 2p_1 + p_4 + p_7 + p_8 + p'_1 + p'_2 + p'_4 - p'_5 + p'_7 + p'_8$

t_1 at M_7 is fired and the resulting marking is $M_{15} = p_1 + p_2 + p_6 + p_8 + 2p'_2 - p'_5 + p'_6 + p'_8$

t_1^+ at M_3 is fired and the resulting marking is $M_{16} = 2p_1 + p_4 + p_5 + 2p_8 + 2p_2' + p_4' - p'_5 + 2p_8'$

t_1^+ at M_7 is fired and the resulting marking is $M_{17} = 2p_1 + p_5 + p_6 + p_8 + 2p'_2 - p'_5 + p'_6 + p'_8$

t_1^+ at M_6 is fired and the resulting marking is $M_{18} = 2p_1 + p_4 + p_7 + p_8 + p'_1 + p'_2 + p'_4 - p'_5 + p'_7 + p'_8$

t_1^+ at M_{10} is fired and the resulting marking is $M_{19} = 2p_1 + p_6 + p_7 + p_1' + p'_2 - p'_5 + p'_6 + p_7'$

t_1^+ at M_5 is fired the resulting marking is $M_{20} = p_1 + p_2 + p_6 + p_8 + p_{11} - p'_1 + 2p'_2 - p'_5 + p'_6 + p'_8$

t_1^+ at M_1 is fired and the resulting marking is $M_{21} = p_1 + p_2 + p_4 + 2p_8 + 2p'_2 + p'_4 - p'_5 + 2p'_8$

t_1^+ at M_9 is fired and the resulting marking is $M_{22} = p_1 + p_3 + p_5 + 2p_8 - p'_1 + 2p'_2 + p'_3 - p'_5 + 2p'_8$

t_1^+ at M_8 is fired and the resulting marking is $M_{23} = p2 + p_3 + 2p_8 - p'_1 + 2p'_2 + p'_3 - p'_5 + 2p'_8$

All these states are included in the ERG as shown below:

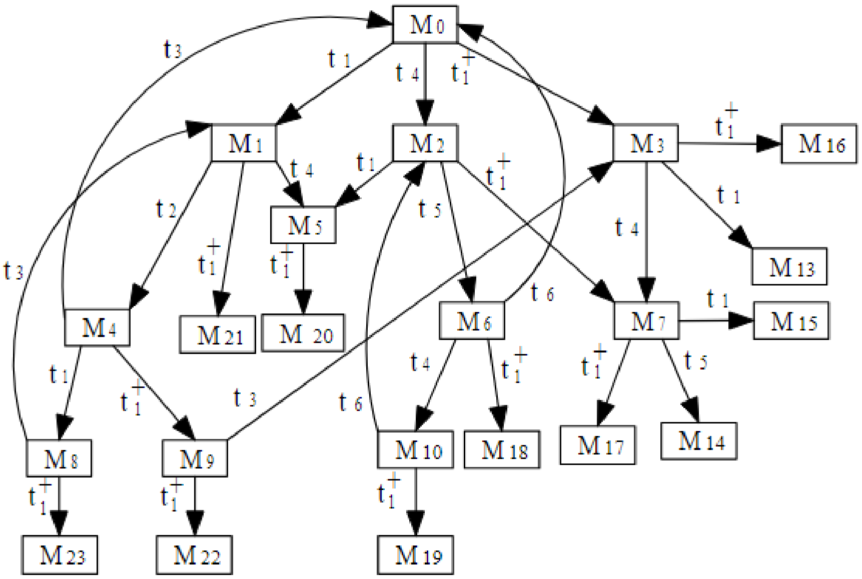

Formulate the ILPP as follows:

```
Min=M;
M>=b;
M+y1-z1>=b;
M+y4-z4>=b;
M+y1-z1+y2-z2>=b;
M+y1-z1+y4-z4>=b;
M+y4-z4+y5-z5>=b;
M+2*y1-2*z1+y2-z2>=b;
M+2*y4-2*z4+y5-z5>=b;
y1-z1+y2-z2+y3-z3=0;
y4-z4+y5-z5+y6-z6=0;
M+2*y1-2*z1<=b-1;
M+y1-z1+y4-z4+y5-z5<=b-1;
M+2*y1-2*z1+y4-z4<=b-1;
M+y1-z1+2*y4-2*z4+y5-z5<=b-1;
M+3*y1-3*z1+y2-z2<=b-1;
@gin(y1);
```

```
@gin(z1);
@gin(y2);
@gin(z2);
@gin(y3);
@gin(z3);
@gin(y4);
@gin(z4);
@gin(y5);
@gin(z5);
@gin(y6);
@gin(z6);
@gin(M);
@gin(b);
end
```

The ILPP is solved in the software Lingo as shown in the following figure.

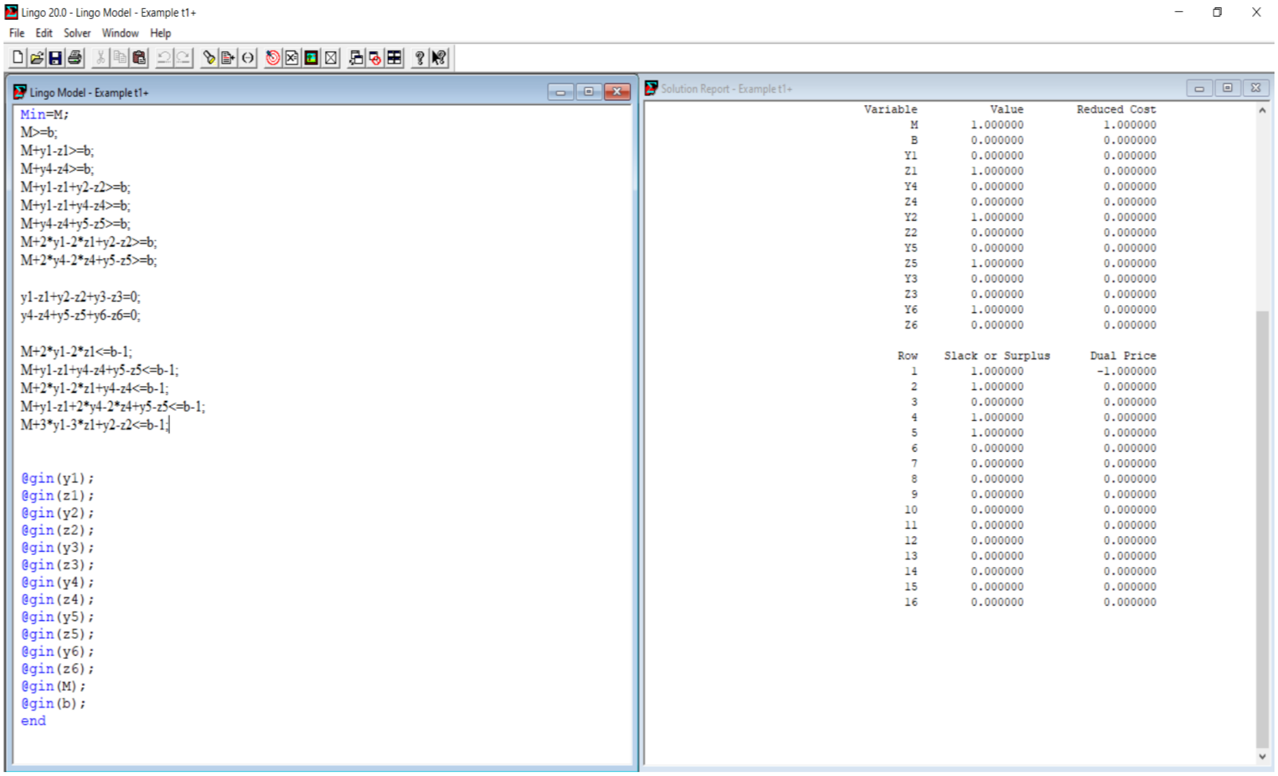

Example 3: The observation system $(\mathcal{N}'', M_0')$ as shown in the figure below, we will study the states of the system step by step as follows:

The system starts with the initial marking $M_0 = 2p_1 + 2p_5 + 2p_6 + 2p_1' + 2p_5' + 2p'_6$

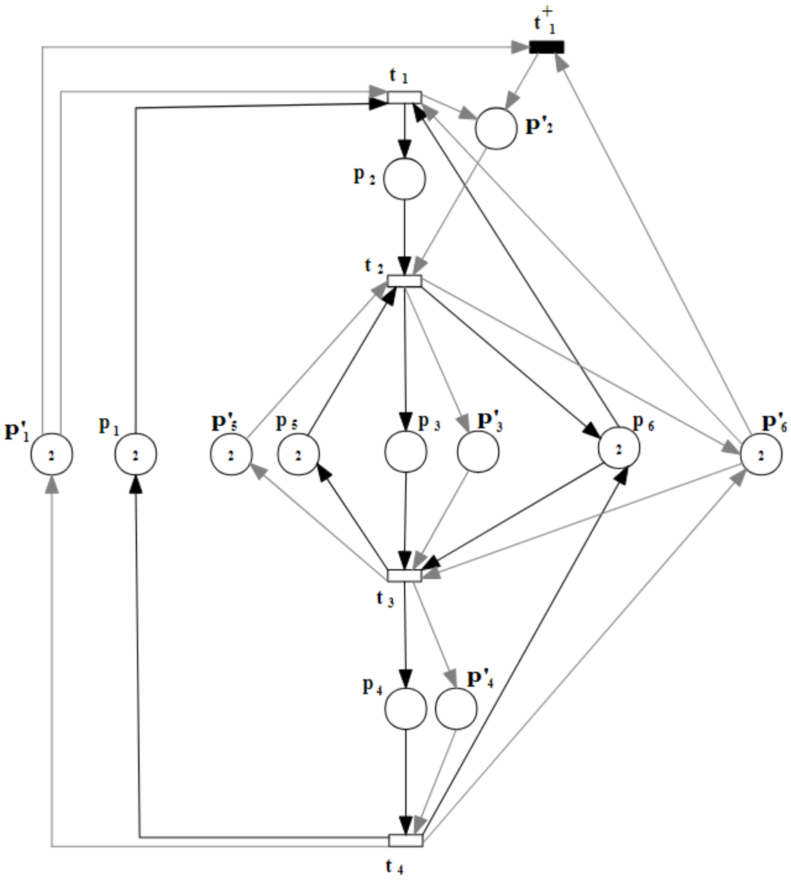

t_1 at M_0 is fired and the resulting marking is $M_1 \ = p_1 + p_2 + 2p_5 + p_6 + p'_{\ 1} + p'_{\ 2} + 2p'_{\ 5} + p'_{\ 6}$

t_1^+ at M_0 is fired and the resulting marking is $M_2 \ = 2p_1 + 2p_5 + 2p_6 + p'_{\ 1} + p'_{\ 2} + 2p'_{\ 5} + p'_{\ 6}$

t_1 at M_1 is fired and the resulting marking is $M_3 \ = 2p_2 + 2p_5 + 2p'_{\ 2} + 2p'_{\ 5}$

t_2 at M_1 is fired and the resulting marking is $M_4 \ = p_1 + p_3 + p_5 + 2p_6 + p'_{\ 1} + p'_{\ 3} + p'_{\ 5} + 2p'_{\ 6}$

t_1^+ at M_1 or t_1 at M_2 is fired and the resulting marking is $M_5 \ = p_1 + p_2 + 2p_5 + p_6 + 2p'_{\ 2} + 2p'_{\ 5}$

t_1^+ at M_2 is fired and the result is $M_6 \ = 2p_1 + 2p_5 + 2p_6 + 2p'_{\ 2} + 2p'_{\ 5}$

t_1 at M_4 or t_2 at $M_3$is fired and the resulting marking is $M_7 \ = p_2 + p_3 + p_5 + p_6 + p'_{\ 2} + p'_{\ 3} + p'_{\ 5} + p'_{\ 6}$

t_3 at M_4 is fired and the resulting marking is $M_8 = p_1 + p_4 + 2p_5 + p_6 + p'_{\ 1} + p'_{\ 4} + 2p'_{\ 5} + p'_{\ 6}$

t_1^+ at M_4 or t_2 at M_5 is fired and the resulting marking is $M_9\ = p_1 + p_3 + p_5 + 2p_6 + p'_2 + p'_3 + p'_5 + p'_6$

t_2 at M_7 is fired and the resulting marking is $M_{10}\ = 2p_3 + 2p_6 + 2p'_3 + 2p'_6$

t_1 at M_8 or t_3 at M_7 is fired and the resulting marking is $M_{11}\ = p_2 + p_4 + 2p_5 + p'_2 + p'_4 + 2p'_5$

t_3 at M_9 or t_1^+ at M_8 is fired and the resulting marking is $M_{12}\ = p_1 + p_4 + 2p_5 + p_6 + p'_2 + p'_4 + 2p'_5$

t_3 at M_{10} or t_2 at M_{11} is fired and the resulting marking is $M_{13}\ = p_3 + p_4 + p_5 + p_6 + p'_3 + p'_4 + p'_5 + p'_6$

t_3 at M_{13} is fired and the resulting marking is $M_{14}\ = 2p_4 + 2p_5 + 2p'_4 + 2p'_5$

t_1 at M_5 or t_1^+ at M_3 is fired and the resulting marking is $M_{15}\ = 2p_2 + 2p_5 - p'_1 + 3p'_2 + 2p'_5 - p'_6$

t_1^+ at M_5 or t_1 at M_6 is fired and the resulting marking is $M_{16}\ = p_1 + p_2 + 2p_5 + p_6 - p'_1 + 3p'_2 + 2p'_5 - p'_6$

t_1^+ atM_6 is fired and the resulting marking is $M_{17}\ = 2p_1 + 2p_5 + 2p_6 - p'_1 + 3p'_2 + 2p'_5 - p'_6$

t_1^+ at M_7 or t_1 at M_9 is fired and the result is $M_{18}\ = p_2 + p_3 + p_5 + p_6 - p'_1 + 2p'_2 + p'_3 + p'_5$

t_1^+ at M_9 is fired and the resulting marking is $M_{19}\ = p_3 + p_5 + 2p_6 - p'_1 + 2p'_2 + p'_3 + p'_5$

t_1^+ at M_{10} is fired the resulting marking is $M_{20} =\ 2p_3 + 2p_6 - p'_1 + p'_2 + 2p'_3 + p'_6$

t_1^+ at M_{11} or t_1 at M_{12} is fired and the resulting marking is $M_{21}\ = p_2 + p_4 + 2p_5 - p'_1 + 2p'_2 + p'_4 + 2p'_5 - p'_6$

t_1^+ at M_{12} is fired and the resulting marking is $M_{22}\ = p_1 + p_4 + 2p_5 + p_6 - p'_1 + 2p'_2 + p'_4 + 2p'_5 - p'_6$

t_1^+ at M_{13} is fired and the resulting marking is $M_{23}\ = p_3 + p_4 + p_5 + p_6 - p'_1 + p'_2 + p'_3 + p'_4 + p'_5$

t_1^+ at M_{14} is fired and the resulting marking is $M_{24}\ = 2p_4 + 2p_5 - p'_1 + p'_2 + 2p'_4 + 2p'_5 - p'_6$

All these states are included in the ERG as shown below:

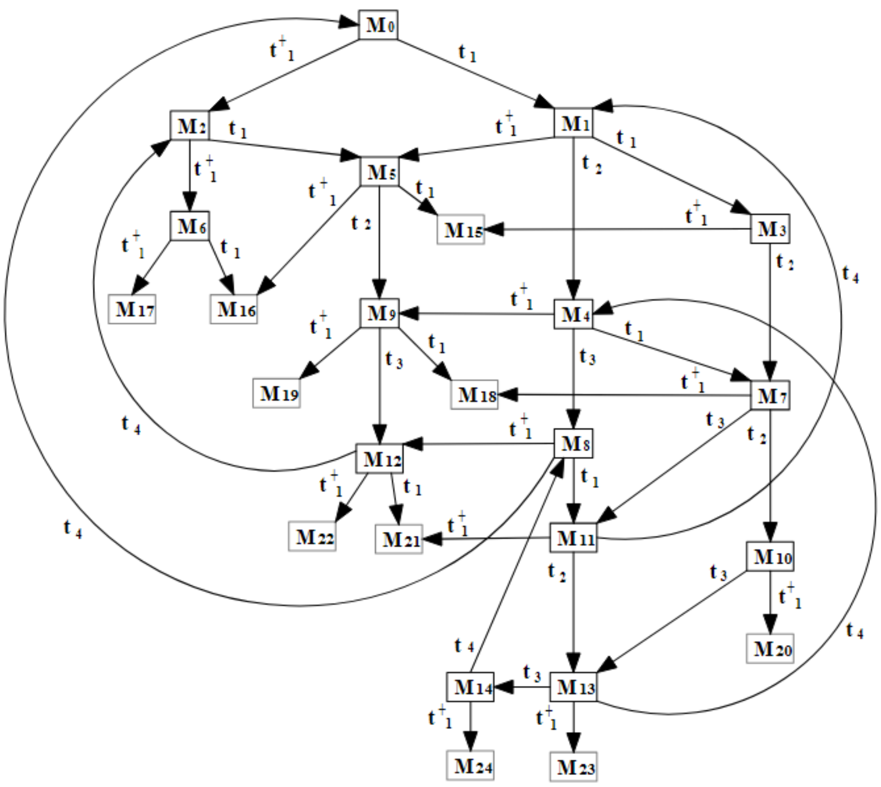

Formulate the ILPP as follows:

```
Min=M;
M>=b;
M+y1-z1>=b;
M+2*y1-2*z1>=b;
M+y1-z1+y2-z2>=b;
M+2*y1-2*z1+y2-z2>=b;
M+y1-z1+y2-z2+y3-z3>=b;
M+2*y1-2*z1+2*y2-2*z2>=b;
M+2*y1-2*z1+y2-z2+y3-z3>=b;
M+2*y1-2*z1+2*y2-2*z2+y3-z3>=b;
M+2*y1-2*z1+2*y2-2*z2+2*y3-2*z3>=b;
y1-z1+y2-z2+y3-z3+y4-z4=0;
M+3*y1-3*z1<=b-1;
M+3*y1-3*z1+y2-z2<=b-1;
M+3*y1-3*z1+2*y2-2*z2<=b-1;
M+3*y1-3*z1+y2-z2+y3-z3<=b-1;
M+3*y1-3*z1+2*y2-2*z2+y3-z3<=b-1;
M+3*y1-3*z1+2*y2-2*z2+2*y3-2*z3<=b-1;
@gin(y1);
@gin(z1);
@gin(y2);
@gin(z2);
@gin(y3);
@gin(z3);
@gin(y4);
```

```
@gin(z4);
@gin(M);
@gin(b);
end
```

The ILPP is solved in the software Lingo as shown in the following figure.

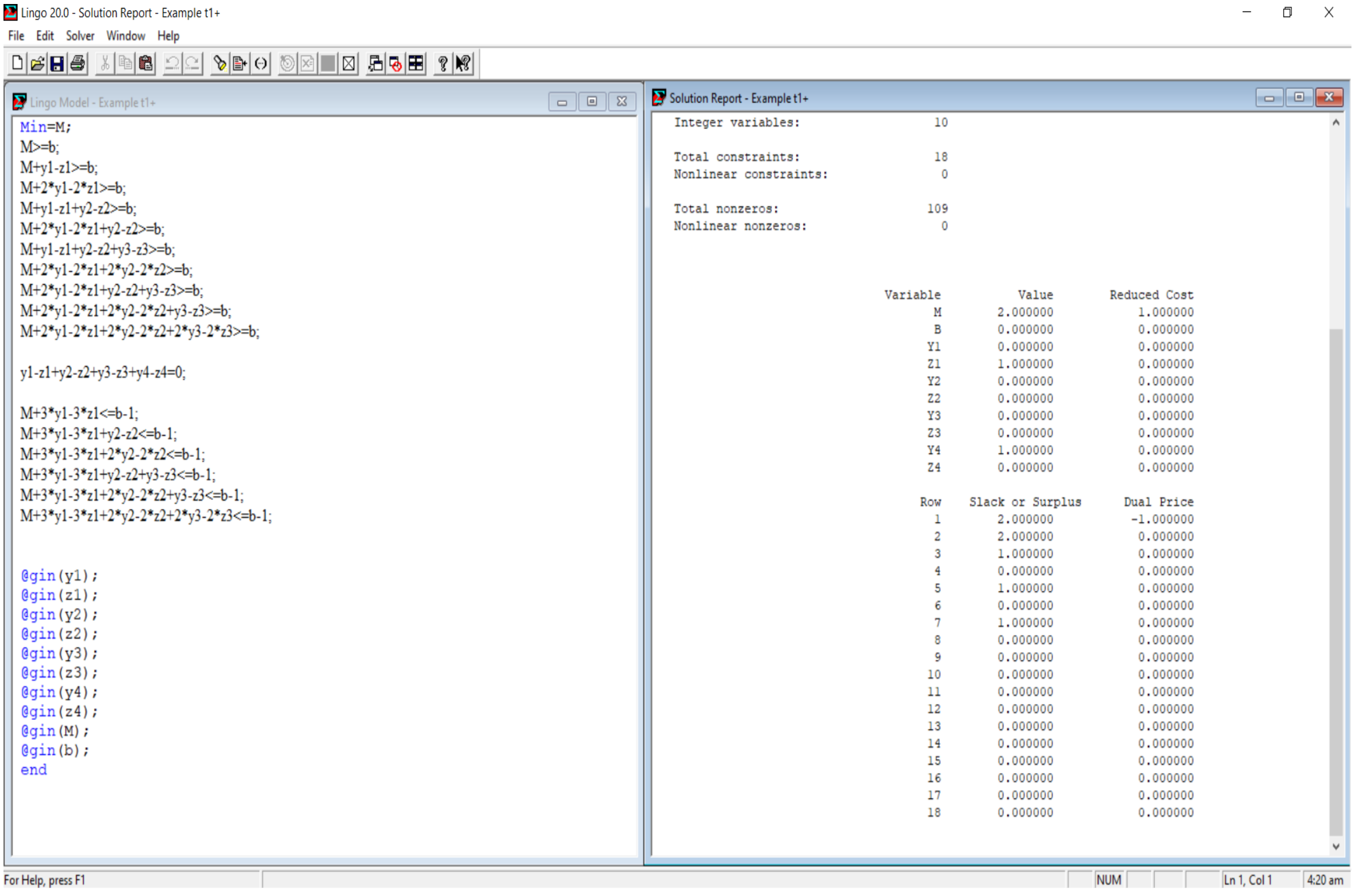

Supplement: S1 Data — (ZIP) [file pone.0314104.s001.zip › Supplementary files (S1).pdf]
